# Increased imaging ligand hydrophilicity and improved pharmacokinetic properties provides enhanced in vivo targeting of fibroblast activation protein

**DOI:** 10.1038/s44303-024-00028-0

**Published:** 2024-08-02

**Authors:** Radhika Narain, Ian Nessler, Paul L. Richardson, Jamie E. Erickson, Yuzhen Wang, Jacqueline Ferri, Heather L. Knight, Shaughn H. Bryant, Lucy A. Phillips, Liang Zhang, Soumya Mitra

**Affiliations:** 1 Former AbbVie Employee, Worcester, MA USA; 2 AbbVie Inc., South San Francisco, CA 94080 USA; 3 AbbVie Inc., North Chicago, IL 60064 USA; 4 AbbVie Bioresearch Center, Worcester, MA 01605 USA

**Keywords:** Immunology, Imaging the immune system, Imaging, Microscopy, Sensors and probes

## Abstract

In this work, the impact of physicochemical modifications on pharmacokinetics and in vivo targeting of a small molecule fibroblast activation protein inhibitor (FAPI) imaging ligand in a murine model of rheumatoid arthritis was evaluated. While similar ligands have been well-reported in oncology for molecular imaging and radiotherapy, there are limited reports of FAPI derivatives in targeted applications in immunology. As inflammation may increase both specific and non-specific delivery of targeted agents in general, we sought to identify the optimal targeted molecular imaging probe characteristics for efficient cell surface engagement. A series of FAPI derivatives were synthesized and their physicochemical properties modified via conjugation of fluorescent dyes and/or an albumin-binding small molecule. The impact of these modifications on cell surface binding affinity was assessed using an overexpressing cell line. Additionally, a thorough mechanistic characterization of fibroblast activation protein (FAP) cell surface internalization was evaluated in both overexpressing and endogenously expressing cells. Lastly, the pharmacokinetics and in vivo uptake in inflamed arthritic paws were characterized via near-infrared (NIR) imaging. All targeted molecular imaging agents tested maintained strong nanomolar binding affinity to cell surface FAP independent of chemical modification. The murine fibroblast-like synoviocytes expressed lower absolute cell-surface FAP compared to a transfected line, and the net internalization half-life measured for the transfected cells via flow cytometry was 7.2 h. The unmodified FAPI ligand exhibited the poorest in vivo targeting, likely resulting from its large apparent volume of distribution (62.7 ml) and rapid systemic clearance (*t*_*1*/2_ = 0.5 h). Conjugation of a charged, hydrophilic AF647 fluorophore decreased systemic clearance (*t*_*1*/2_ = 2.1 h) and demonstrated a 2-fold improvement in blocking FAPI-800CW engagement of FAP in vivo when compared to blocking of FAPI-800CW with FAPI with up to 2.8-fold improvements noted for the equivalent albumin binding construct comparison.

## Introduction

Rheumatoid arthritis (RA) is an autoimmune disorder characterized by chronic inflammation of synovial joints leading to cartilage and bone destruction^1^. Early diagnosis and treatment of RA can avert or substantially slow progression of joint damage in up to 90% of patients, thereby preventing irreversible disability^2^. Current RA treatments include anti-inflammatory drugs, anti-rheumatic drugs, biological agents, or corticosteroids that are primarily palliative treatments and may take several months to induce response^3,4^. Due to delayed response and irreversibility of the disease, enabling early diagnosis may better allow for curative intervention in RA or treatments with improved outcomes^5,6^. However, current diagnoses rely on systemic biomarkers, which lack adequate sensitivity and specificity to provide insight into local joint conditions or identify at-risk individuals. Other methods, such as ultrasonography and magnetic resonance imaging (MRI), encounter separate challenges. While ultrasonography may adequately assess synovitis, detection of arthritis in early stages is sensitivity limited. In contrast, MRI can detect joint inflammation and disease progression at an early stage but can be resource intense for routine screening^7,8^. Targeted molecular imaging has the potential to address these limitations and detect joint inflammation in a minimally invasive manner^9,10^. Towards that end, researchers are exploring a variety of targeted agents for RA, including extracellular matrix proteins and cell-surface receptors^11–13^.

Activated synovial fibroblasts and immune cells produce inflammatory cytokines, which are hypothesized to drive RA development and progression^14,15^. While imaging immune cell activity may correlate with clinical disease severity, there is growing interest that direct targeting of activated synovial fibroblasts may improve imaging sensitivity and enable/complement orthogonal approaches such as targeted drug delivery. Fibroblast activation protein (FAP) is a 97 kDa type II transmembrane serine protease with elevated expression in reactive stromal fibroblasts found in cancer, arthritis, fibrosis, and interstitial lung disease^16–22^. FAP upregulation is also observed in pathogenic populations of activated synovial fibroblasts in the inflamed joints of RA patients^23,24^. In arthritic joints, FAP is predominantly expressed by activated fibroblast-like synoviocytes in joint cavities and is implicated in key effector roles during disease progression both preclinically and clinically^15,25^. The experimental deletion of these fibroblasts in mouse models of inflammatory arthritis is sufficient to attenuate synovial inflammation and bone erosion, highlighting the potential of FAP-targeting molecules as therapeutics in addition to imaging agents^24,26–29^.

While antibody-based molecular imaging strategies for FAP have been investigated, their slow clearance rate led to high background signal and limited sensitivity in preclinical models of RA^30,31^. More recently, a variety of quinoline-based^32,33^ FAP inhibitors (FAPIs) have shown promising results for both targeted molecular imaging and drug delivery applications in oncology^34–39^. While cleared rapidly via renal elimination, these lower molecular ligands have several possible advantages over antibodies, including improved tissue penetration^40,41^, lower immunogenicity^42^, and reduced cost of goods^43^. While high affinity small molecule ligands for cell surface receptors are rare, the reported single digit nanomolar affinity for FAPI derivatives suggest high imaging contrast can be achieved following rapid antigen binding and efficient washout^40^.

While the popularity of the FAPI probes has surged for radiopharmaceutical development in oncology^44,45^, targeted strategies for FAP in immunology are less common, despite reported expression in RA and uptake of ^68^Ga-FAPI-04 in arthritic joints of a single RA patient^46^. A clinical imaging pilot study evaluated the sensitivity of the positron emission tomography (PET) tracer to assess disease activity in 20 RA patients and reported positive correlation between maximum standardized uptake values (SUV) in joints and clinical radiographic biomarkers^47^. In a recent study, ^68^Ga-FAPI-04 PET was performed in 120 patients with inflammatory joint disease, and focal FAP-tracer accumulation at synovial and entheseal sites in patients with arthritis but not in non-arthritic controls^48^. Another new report used FAPI-PET imaging to assess arthritic joint remodeling in a RA patient in response to blinatumomab, a CD19xCD3 Bispecific T cell engager^49^. These elegant studies demonstrate that FAP tracers may be used as an imaging biomarker to monitor mesenchymal changes and disease activity in arthritic patients. While these early reports are encouraging, there is limited information on rational molecular design of FAP tracers for applications outside oncology. Imaging agent properties such as affinity and clearance may also be impacted by the local physiology of diseases tissue. For example, delivering agents to inflamed tissues presents unique challenges such as increased non-specific uptake and often, reduced levels of total antigen for immune cell-specific targets. Thus, we believe an improved understanding of target expression and target biology such as receptor trafficking will help identify the salient probe properties to optimally engage FAP in RA. In this work, we synthesized a series of FAPI ligands varying in physicochemical properties that resulted in diverse clearance rates. These probes were then used to identify imaging agent properties that allow for high imaging contrast and efficient target engagement using a well-documented murine disease model of RA. Through a series of in vitro and in vivo experiments, we investigated the trafficking kinetics and delivery mechanisms of a diverse array of FAPI derivatives. Our findings indicate that FAP targeting can be optimized by incorporating non-albumin binding modifications that enhance the hydrophilicity and pharmacokinetic profile.

## Results

**Fig. 1:**
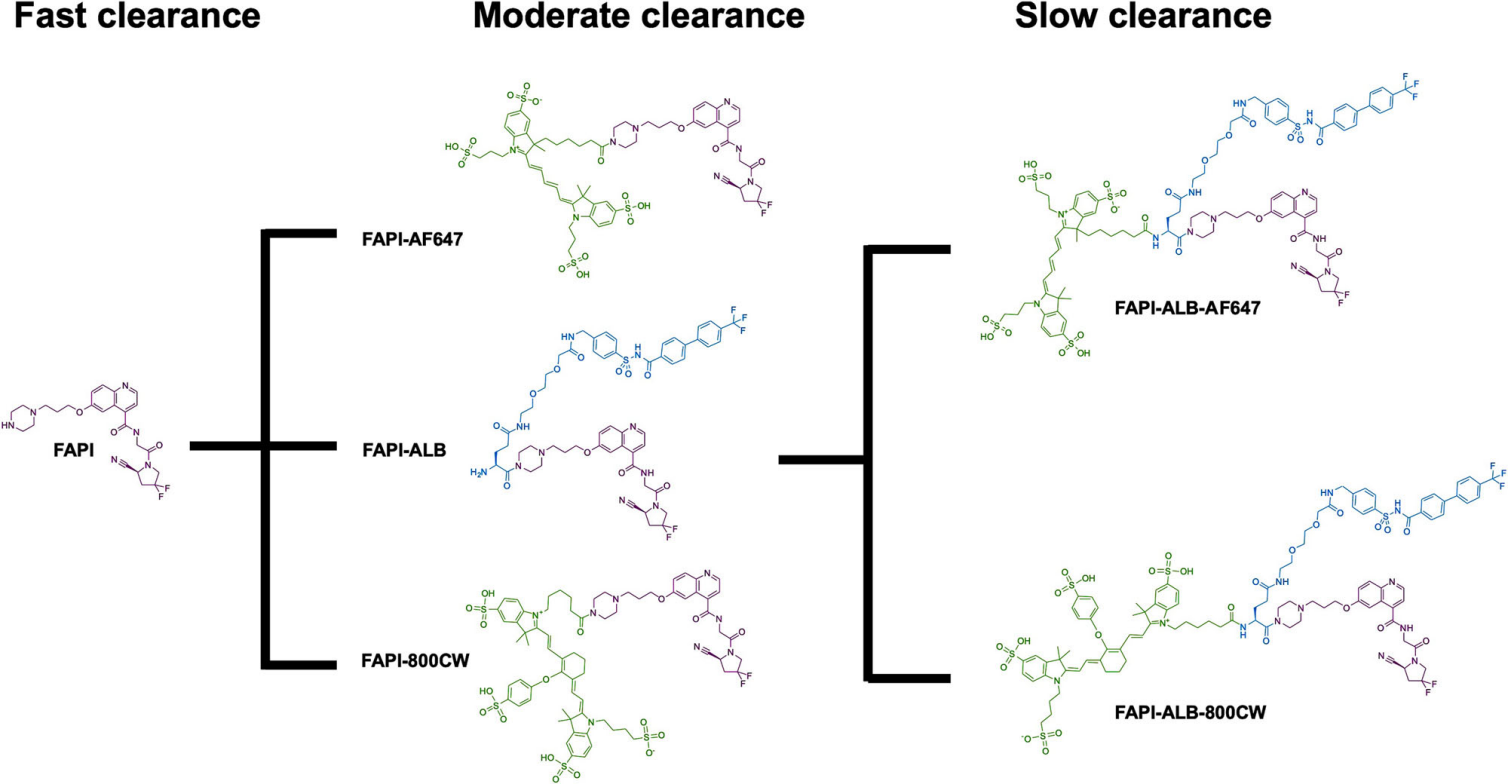
Design and synthesis of various FAPI conjugates. Conjugation to AF488 enables surface signal quench for internalization assays. Conjugation to AF647 enables microscopy and is also used for in vivo competition. Conjugation to 800CW enables near-infrared imaging. Parallel set of probes were designed and evaluated with/without an albumin-binding moiety. Core FAPI structure is highlighted in purple, albumin-binding small molecule in blue, and fluorophores in green.

A series of fluorescent FAPI conjugates were synthesized to understand the impact of internalization and plasma kinetics on in vivo FAP molecular imaging in preclinical arthritis models (Fig. 1). The FAPI derivatives used in our study are analogous to FAPI-04^35,36^. In our synthesis of FAPI-04-based probes, following deprotection of the Boc-piperazine, the probe was reacted with various fluorophores rather than conjugating an amine-reactive DOTA. FAPI-AF488 and FAPI-AF647 probes were synthesized to characterize in vitro receptor internalization kinetics via flow cytometry and microscopy, respectively. Notably, AF488 was selected due to ease of Trypan Blue quench in cytometry applications. For in vivo imaging, selection of the detectable label is not as straight forward. On one hand, lipophilic fluorophores such as non-sulfonated cyanine dyes can impart both albumin-binding properties to slow clearance and enable NIR tracking. However, direct conjugation of Cy7 significantly weakened FAP binding in vitro (data not shown). To maintain binding affinity while also increasing the systemic half-life of FAPI, alternative fluorophores and protein binding constructs were evaluated. The red dye (AF647) and near-infrared (NIR) dye (800CW) conjugates of FAPI designs were prepared^50^ (Fig. 1). The biphenyl acylsulfonamide moiety—with high binding affinity towards domain III of human serum albumin—was introduced to slow plasma half-life, and these synthesized FAPI compounds have an ALB suffix.

Compound lipophilicity was predicted through a cLogP calculation from the molecular structure, whereas plasma protein binding (PPB) was measured experimentally using equilibrium dialysis (Table 1). Whereas FAPI alone had relatively low plasma protein binding (44.7%), conjugation to biphenyl acylsulfonamide significantly increased albumin binding (>98%). Addition of the hydrophilic sulfonated fluorophore AF647 did not substantially change the plasma protein binding relative to the FAPI-ALB binder alone.

**Table 1:** Physicochemical parameters for FAPI conjugates used during in vivo applications, including MW, cLogP and plasma protein binding

Reagents	MW (Da)	cLogP	% Protein Bound ± SD
FAPI	486.51	0.3	44.7 ± 3.1
FAPI-ALB	1177.2	1.0	98.9 ± 0.2
FAPI-AF647	1328.52	−0.3	20 ± 12.7
FAPI-ALB-AF647	2019.21	−1.0	99.7 ± 0.1

Addition of sulfonated dyes alone increases hydrophilicity. Addition of the albumin-binding small molecule significantly increases plasma protein binding.

**Fig. 2:**
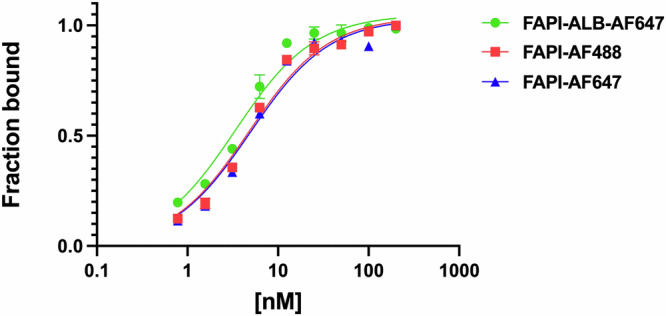
Affinity curves for directly measured FAPI conjugates. Direct detection of fluorescent label suggests all ligands maintain strong binding affinity.

^36^Cell surface affinity data were generated to assess the impact of fluorophore conjugations on binding affinity of the parental FAPI small molecule. While low degree of label modifications minimally impact binding for higher molecular weight protein ligands, the same may not be true for low molecular weight ligands as the label may be proximal to binding pockets. Nine-point affinity curves were generated for AF488 and AF647 FAPI derivatives via flow cytometry using HEK293-hFAP cells (Fig. 2).

Binding affinity (K_D_) for unconjugated and 800CW conjugates were calculated indirectly through competition assays conducted at equimolar concentrations of probes with known binding affinity and unknown binding affinity. All FAPI derivatives maintained low nanomolar K_D_ whether measured directly or indirectly (Table 2A) consistent with previous literature measurements for FAPI-04 EC_50_ through competition assays (6.5 nM)^36^.

**Table 2:** **A** Cell surface determined binding affinities (K_D_) for directly measured and indirectly* calculated FAP conjugates. **B** Flow cytometry using saturating concentrations of FAPI-AF488 and MESF beads to quantify surface expression in overexpressing and endogenous cells

Reagents	K_D_ (nM) ± SEM
A	
FAPI	6*
FAPI-AF488	4.8 ± 0.5
FAPI-AF647	5.0 ± 0.9
FAPI-800CW	8*
FAPI-ALB	3.6*
FAPI-ALB-AF647	3.4 ± 0.4
FAPI-ALB-800CW	9.9*
B	
Cell line	Receptors/cell
RAFLS	~25,000
HEK293-hFAP	~1,000,000

Fluorophore conjugation had minimal impact on the affinity; FAPI-AF488 and FAPI-AF647 had a K_D_ of 4.8 nM and 5 nM, respectively (Table 2A). The K_D_ of FAPI-800CW was measured to be 8 nM, comparable to FAPI-AF488 and FAPI-AF647. Similarly, the addition of the albumin-binding moiety minimally impacted affinity (K_D_ of 4 nM to 10 nM for ALB compounds). Lastly, we estimated cell surface FAP expression using an overexpressing system (HEK293-hFAP cells) as well as primary RAFLS cells. RAFLS cells had ~ 25,000 copies per cell; HEK293-hFAP had ~ 1 million per cell (Table 2B). Both cell types had a negligible amount of non-specific sticking to FAPI ligands (Fig. S1).

**Fig. 3:**
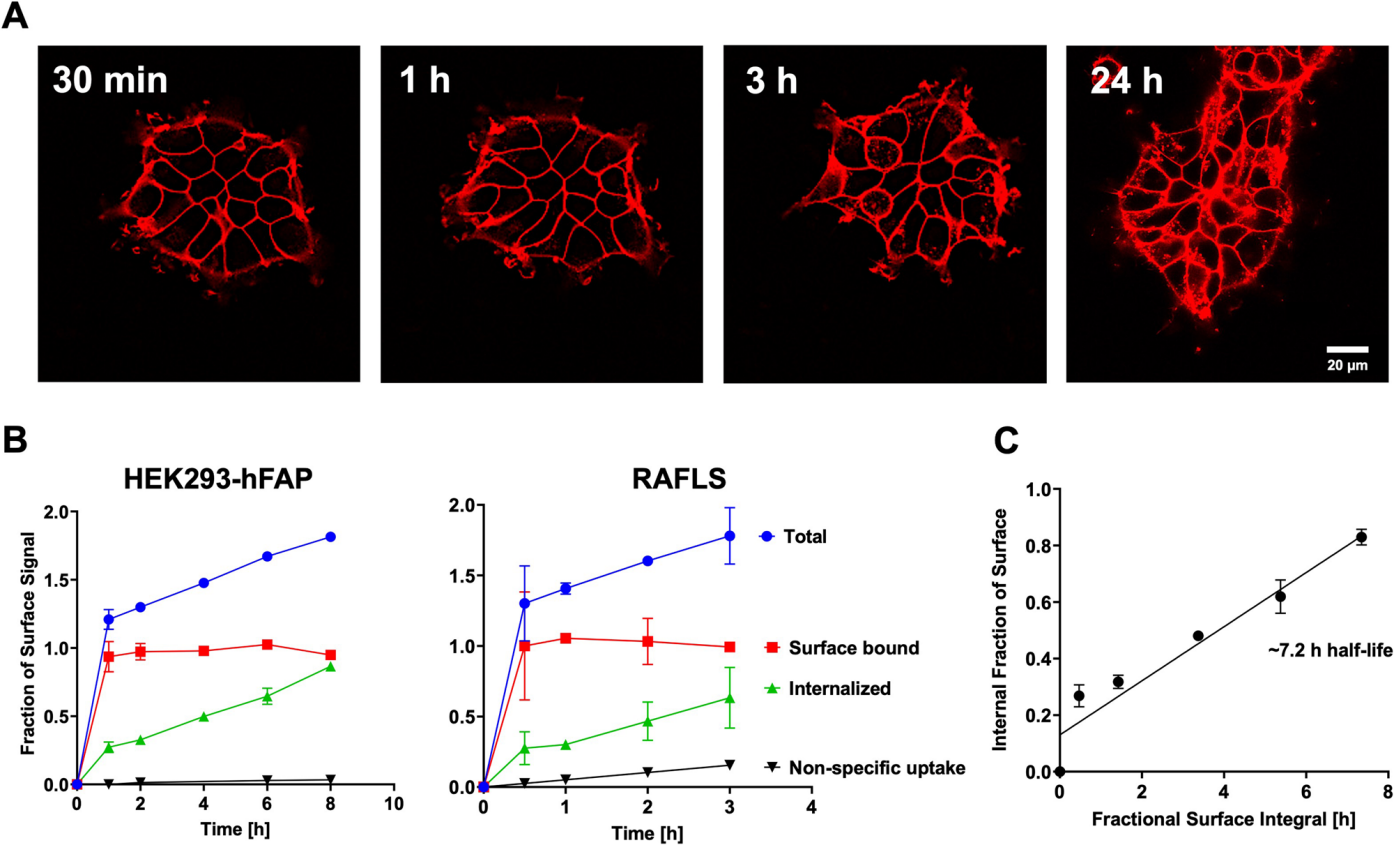
FAP receptor trafficking assessment with live-cell time lapse microscopy and flow cytometry. **A** Live-cell time-lapse of HEK293-hFAP cells labeled with continuous exposure of FAPI-AF647. Surface saturation occurs quickly, followed by appearance of punctate red spots, consistent with receptor trafficking. Scale bar in (**A**) is representative for all images. **B** In vitro internalization assays with overexpressing and endogenous cells recapitulate the microscopy results. **C** Plot of internal fraction of surface saturation vs the fractional surface integral. Here, the slope is the 1^st^-order net internalization rate constant.

As there are reported differences in both the rate and extent of internalization for cell surface FAP in various cells^35,51^, we evaluated receptor internalization using live-cell time-lapse microscopy and quantitative flow cytometry (Fig. 3).

Qualitatively, live-cell time-lapse imaging of HEK293-hFAP cells showed rapid surface binding upon FAPI addition, followed by receptor mediated endocytosis. Cell surface binding equilibrated rapidly, which agrees well with the strong affinity of the ligand—namely fast k_on_— and the concentration of ligand added in the microscopy experiments (Fig. 3A). At longer times, the punctate, intracellular AF647 signal co-localizes well with lysosomal markers (Fig. S2), suggesting that FAPI is eventually trafficked to lysosomes. Following visual confirmation of endocytosis, we sought to quantify the net internalization rate constant using a modified protocol from Schmidt et al.^52^. The net internalization rate constant was measured on overexpressing HEK293-hFAP cells and endogenously expressing RAFLS cells. Cells were allowed to internalize continuously for predetermined amounts of time and the total intensity per cell was compared to the surface quenched intensity per cell. Importantly, this approach to quantifying the net uptake rate accounts for time-dependent cell-surface expression. After accounting for the quenching efficiency, the fluorescent intensities from total, surface, and internal cellular compartments were plotted against time for the overexpressing and endogenous cells (Fig. 3B). Surface-bound signal remained constant after 1 h, suggesting limited modulation of surface FAP expression during the time course of the experiment. Additionally, near constant surface expression was observed with an AF647 conjugated anti-FAP antibody (Fig. S3). Non-specific uptake was assessed for this assay and showed minimal uptake (<0.2% of internal signal) for HEK293-hFAP cells, while RAFLS cells showed increased non-specific uptake (~20% of internal signal). The internalized signal adjusted for non-specific uptake was plotted against the integral of the surface signal over time and fit via linear regression with the slope being equivalent to the net internalization rate constant (Fig. 3C). The net internalization half-lives for RAFLS and HEK293-hFAP cells were estimated at 3.7 h and 7.2 h, respectively. As primary RAFLS cells are collected from various donors, we conducted additional net internalization assays to measure donor variability. Interestingly, the estimated net internalization half-lives ranged from 3.7 h to 18.2 h (Fig. S4) with differences in non-specific uptake as well. While the absolute flow cytometry signals for donor cell experiments were low, our results highlight challenges for conducting these quantitative assays in low-expressing and/or primary cells.

**Fig. 4:**
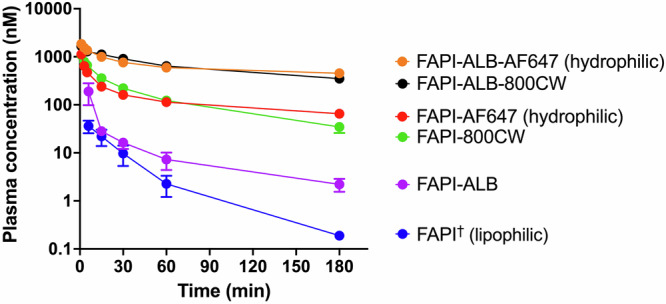
Plasma pharmacokinetics of FAPI reagents in healthy male DBA1/J mice following an IV bolus. ^†^Due to an expectation of rapid clearance, FAPI was dosed in ~25-fold molar excess of other conjugates to maintain plasma concentrations above lower limit of quantification (LLOQ) throughout sampling period. Concentrations plotted have been linearly dose adjusted for comparison to all other probes dosed at 2 nmol.

The moderate internalization half-life measured for FAP and single digit nanomolar affinity of the FAPI probes highlights their potential for target engagement, however, an understanding of systemic exposure in vivo is important to characterize imaging probe capabilities. The impact of FAPI conjugations on pharmacokinetics (PK) was investigated in healthy mice (Fig. 4) and then PK parameters were generated with non-compartmental analysis (Table 3).

**Table 3:** Summary of PK parameters derived from non-compartmental analysis (Mean ± SEM)

Reagents	C_0_ (nM)	Terminal t_1/2_ (h)	AUC_0-inf_ (nM*h)	V_D,ss_ (ml)	Cl (ml/h)
FAPI^a^	50 ± 7	0.5 ± 0.1	14 ± 4	62.7 ± 18.4	234 ± 98
FAPI-ALB	189 ± 52	0.9 ± 0.0	88 ± 18	14.5 ± 8.4	25.2 ± 6.2
FAPI-AF647	1488 ± 30	2.1 ± 0.2	626 ± 46	8.0 ± 0.5	3.23 ± 0.23
FAPI-800CW	1252 ± 52	1.0 ± 0.1	525 ± 25	3.9 ± 0.2	3.83 ± 0.19
FAPI-ALB-AF647	2028 ± 113	5.6 ± 0.1	7326 ± 162	1.3 ± 0.1	0.27 ± 0.01
FAPI-ALB-800CW	1835 ± 129	5.3 ± 0.1	6104 ± 190	1.4 ± 0.1	0.33 ± 0.01

^a^FAPI was delivered at a higher dose than other conjugates to maintain concentration above LLOQ during sampling. Concentration data for this probe has been linearly dose normalized to 2 nmol for consistency to other probes.

Unconjugated FAPI demonstrated the fastest clearance rate with a moderate volume of distribution at steady-state, while conjugation of biphenyl acylsulfonamide, AF647, or 800CW resulted in decreased clearance rates and increased exposures. Addition of biphenyl acylsulfonamide further decreased clearance rates of both fluorescently labeled FAPI derivatives resulting in a terminal clearance half-life of ~5.5 h. In addition to an improved half-life, the fluorescent ALB conjugates demonstrated greater maximum plasma concentrations consistent with lower volumes of distribution, while the rapidly clearing conjugates had moderately lower maximum concentrations associated with larger volumes of distribution (Fig. 4, Table 3).

Given the significant differences in clearance between FAPI derivatives, yet comparable in vitro cell surface affinity, all FAPI derivatives were assessed via in vivo near-infrared imaging in a murine model of RA.

**Fig. 5:**
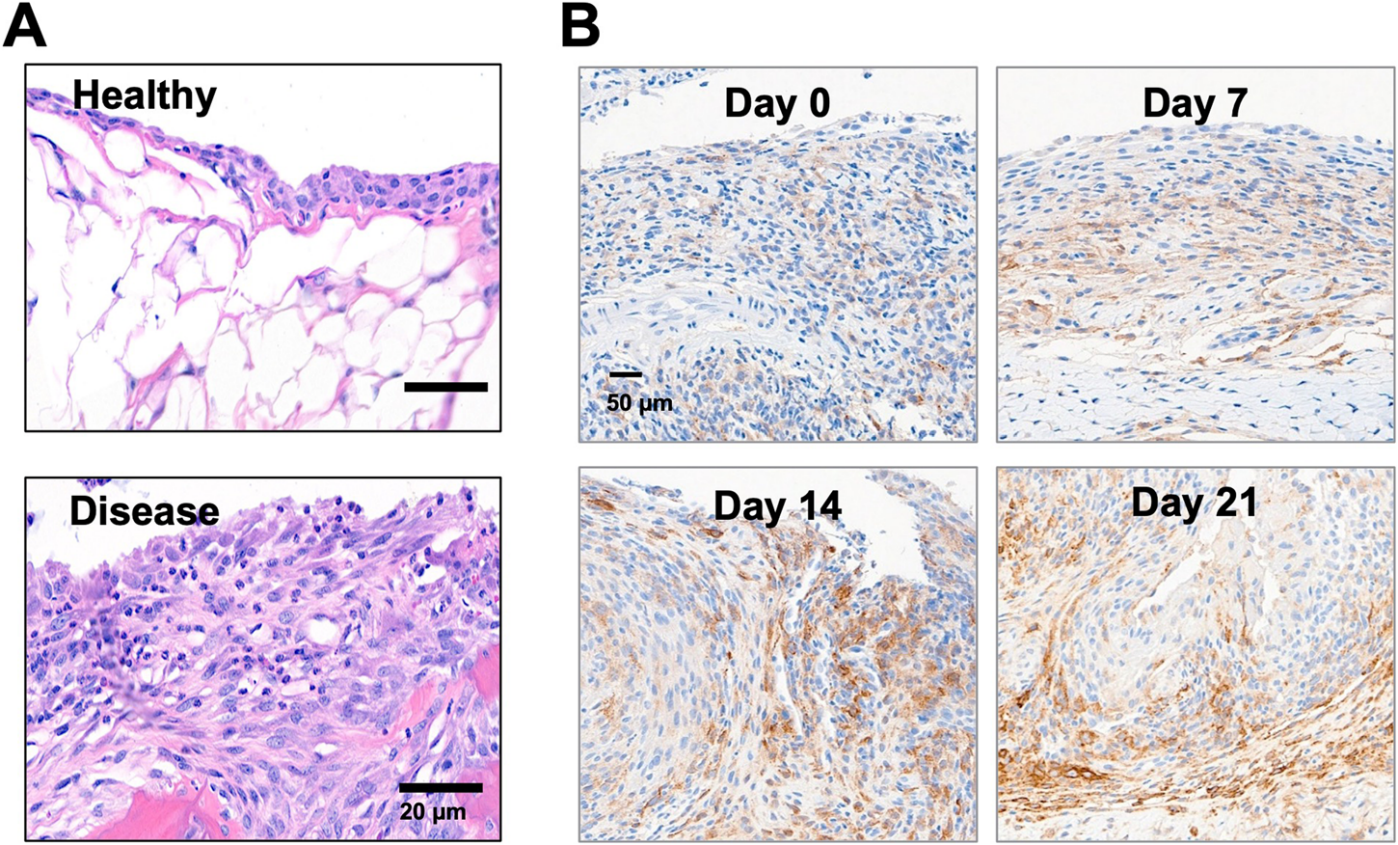
Immunohistochemistry of FAP expression in healthy and diseased murine knee synovial tissue. **A** Enhanced pannus formation in CIA mice compared to healthy mice. **B** Increased FAP expression with increasing disease severity in CIA mice as assessed with IHC. Scale bars in (**A**) and (**B**) are representative for all images in (**A**) and (**B**), respectively.

The CIA murine model of RA was used to evaluate in vivo molecular imaging of FAP preclinically. After the first symptoms of disease^53,54^, paw tissues were collected at 7 d, 14 d, and 21 d with corresponding IHC to confirm disease progression (Fig. 5).

**Fig. 6:**
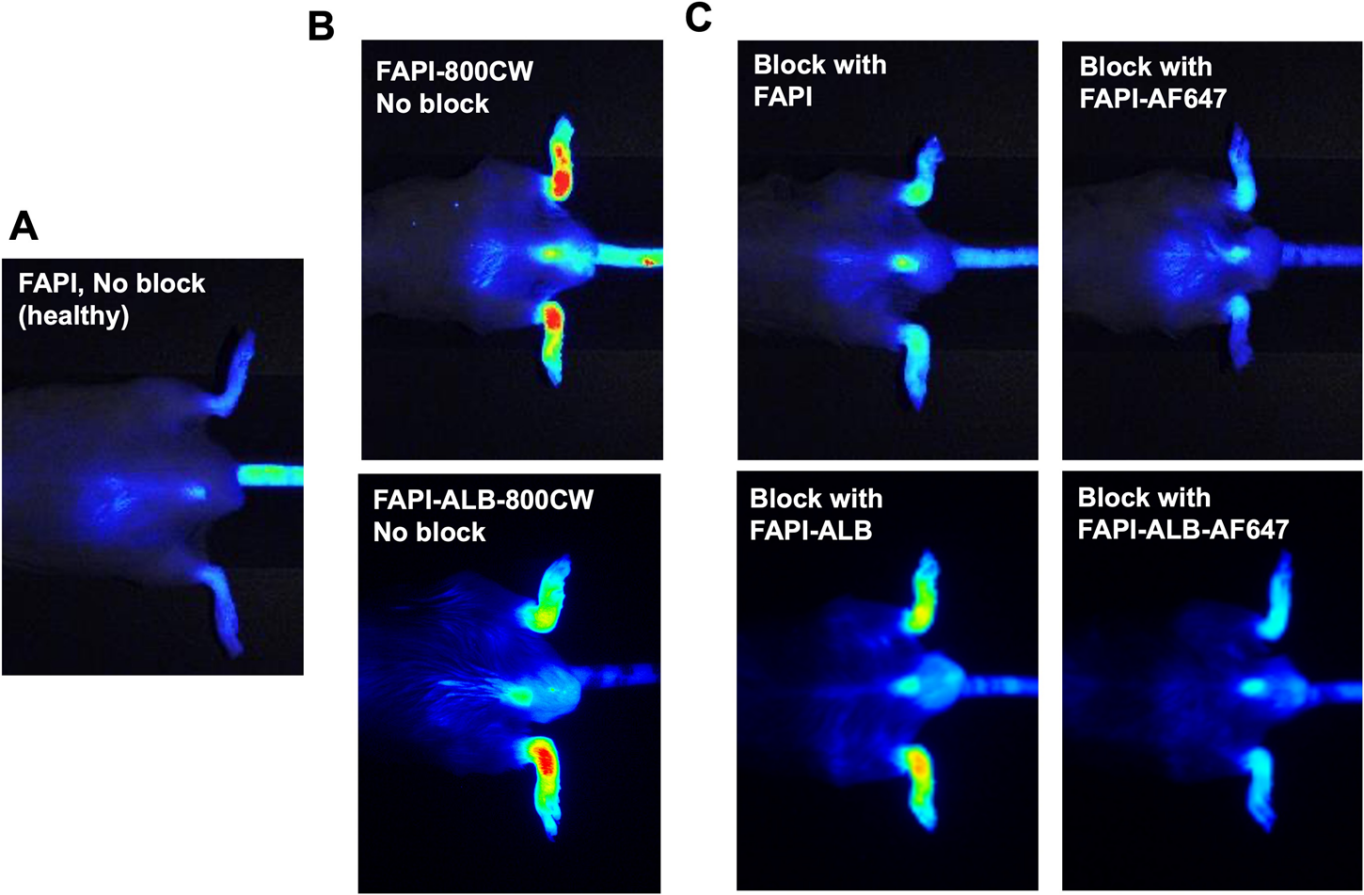
Representative in vivo images of FAPI-800CW and FAPI-ALB-800CW uptake in mouse paws following a 1 nmol IV bolus. Images are shown with identical thresholding. **A** In vivo image of FAPI-800CW in healthy paws. **B** Inflamed paws post dosing with FAPI-800CW at 5 h (top) and FAPI-ALB-800CW at 24 h (bottom). Here, the images represent the maximum NIR signal without competition. **C** Reduced 800CW signal observed in inflamed paws of CIA mice when blocked with FAPI-AF647 (top) or FAPI-ALB-AF647 (bottom). Blocking doses were co-administered with a 50-fold molar excess. Here, greater reductions in NIR signal represent improved targeting of the blocking agent.

Enhanced pannus formation was observed at 21 d vs healthy controls (Fig. 5A). Immunohistochemistry (IHC) suggests increasing FAP expression from 7 d–21 d and correlated with disease severity (Fig. 5B). The IHC signal was evaluated by a board-certified pathologist and found to align with mRNA expression in the inflamed synovium and is predominantly located in the spindle shaped-activated fibroblasts. The specificity of the IHC immunostaining was confirmed in FAP overexpressing HEK293 cell pellets (Fig. S5). After confirming the expression of FAP in this murine model, the FAPI probes were assessed via near-infrared imaging (Fig. 6). As it can be challenging to compare in vivo fluorescent intensities for different fluorophores, the compounds in this work were evaluated based on their ability to block and compete the near-infrared tagged FAPI ligands through co-administration of compounds, rather than imaging each standalone probe in their respective fluorescent channels. The detectable NIR probe was paired with a blocking probe with similar albumin binding capabilities; for example, probes (FAPI and FAPI-AF647) without the albumin-binding small molecule were used to block FAPI-800CW whereas the compounds with albumin-binding small molecule (FAPI-ALB and FAPI-ALB-AF647) were used to block FAPI-ALB-800CW.

**Fig. 7:**
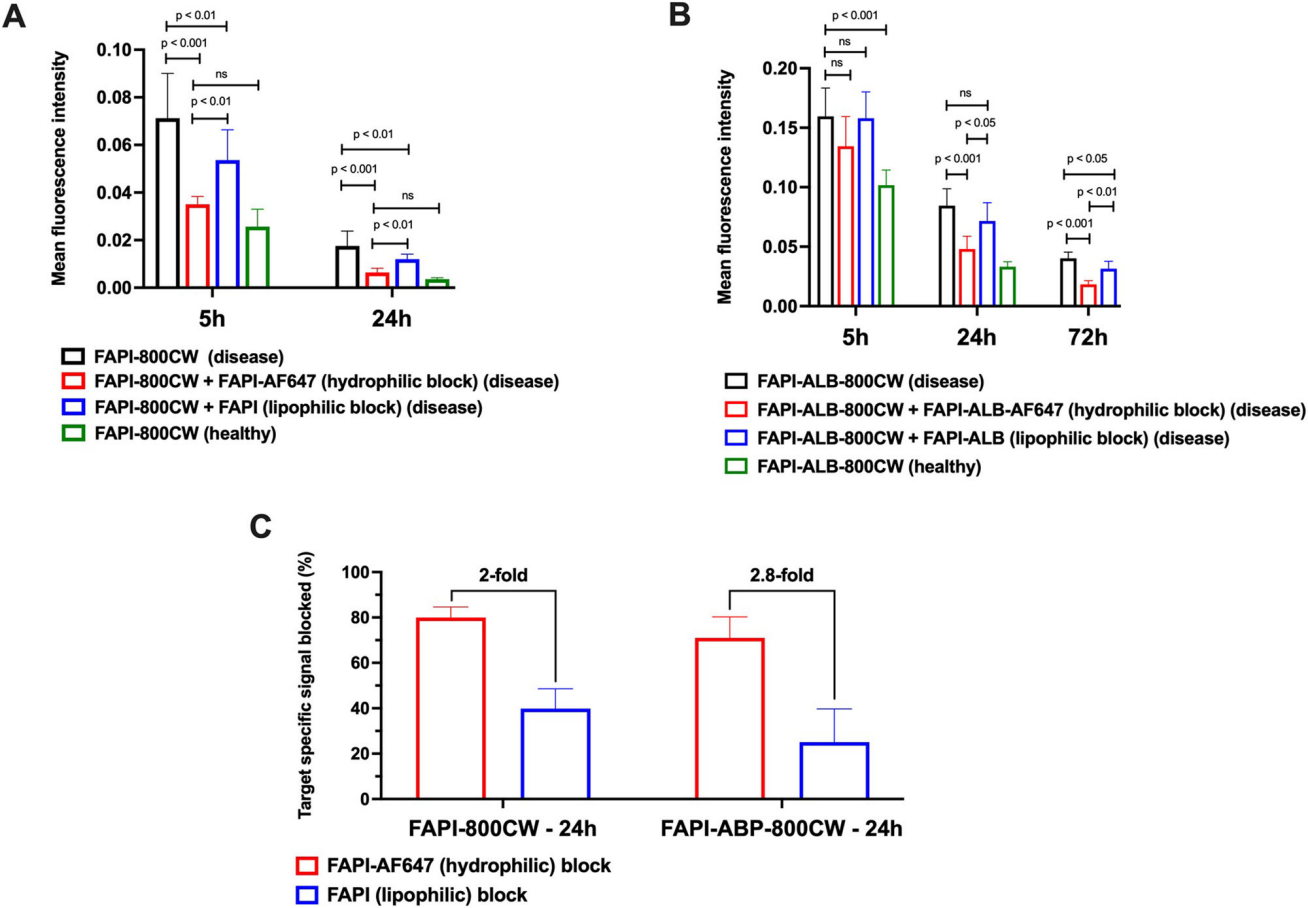
Uptake of FAPI in in healthy and inflamed murine paws. **A** Greater reductions in diseased paw mean fluorescent intensities (MFI) suggest FAPI-AF647 is a better blocker and stronger in vivo FAP engager than FAPI alone. **B** This trend is also observed with addition of the albumin-binding small molecule. Absolute MFI is also higher, likely due to increased perfusion-driven signal. Slower clearance rates also enable imaging out to longer times, but also lead to increased non-specific signal in healthy paws compared to that observed with FAPI. **C** After background subtraction, FAPI-AF647 was demonstrated to block > 2-fold more target specific signal when compared to FAPI for non-albumin (left) and albumin binding (right) versions of FAPI-800CW at 24 h (Mean +/- SEM). ns: non-significant.

FAPI-800CW and FAPI-ALB-800CW uptake in inflamed joints following intravenous (IV) dosing was observed in all groups. Unsurprisingly, the highest 800CW intensity was observed in animals that did not receive blocking doses and the lowest intensity was observed in healthy paws (Fig. 7, Fig. S6). When FAPI-800CW was co-dosed with a molar excess of FAPI-AF647, FAPI-800CW uptake in inflamed paws was reduced to a similar MFI as healthy imaging controls at 5 h (Fig. 7A). Interestingly, a molar excess dose of the parental FAPI ligand did not provide a similar reduction of FAPI-800CW signal, suggesting improved in vivo targeting properties for FAPI-AF647. A comparison of the target specific signal blocked between the parental FAPI ligand and FAPI-AF647 demonstrated a 2-fold improvement in blocking for the faster clearing probes (Fig. 7C). The analogous comparison with the slower clearing FAPI-ALB probes showed similar trends (Fig. 7B, C). Here, FAPI-ALB-AF647 was a better blocker of FAPI-ALB-800CW than FAPI-ALB (24 h: 0.05 ± 0.01 vs 0.07 ± 0.01 for FAPI-ALB-AF647 block vs FAPI-ALB block, respectively). Expectedly, slower systemic clearance increased overall exposure and led to increased non-specific signal, as demonstrated by a 4-fold increase in mean fluorescence intensity in healthy paws at 5 h (0.026 ± 0.007 vs 0.10 ± 0.01 for FAPI-800CW in healthy vs FAPI-ALB-800CW in healthy, respectively, *P* < 0.001). The significant reduction in FAPI-800CW and FAPI-ALB-800CW signals upon co-administration of the AF647 conjugated versions of FAPI and FAPI-ALB, respectively, suggest improved targeting of the AF647 reagents imparted through a combination of their improved hydrophilicity (Table 1) and enhanced systemic circulation (Fig. 4 and Table 3).

## Discussion

Given their well-documented implication in the inflammatory destruction of cartilage and bone within arthritic joints, fibroblast-like synoviocytes (FLS) have served as an attractive diagnostic and therapeutic target in rheumatoid arthritis^55–57^. More recently, targeted therapies focused on direct fibroblast engagement have seen growing interest, largely due to the development and use of a small molecule fibroblast activation protein inhibitor for cell-surface expressed FAP in oncology radiotherapy and drug delivery applications^45,51,58–60^. Targeted molecular tracers are less common in autoimmune indications, however^58^. Increased blood delivery to sites of disease due to inflammation have resulted in greater reliance on perfusion imaging tracers, such as Indocyanine Green (ICG), rather than targeted probes, which require high absolute cell surface expression for high target-background ratios. Recent work^31^ as well as our internal efforts have identified fibroblast activation protein expression in murine models of arthritis. Here, a high affinity, low molecular weight ligand for cell surface FAP has potential for enhanced contrast.

In this work, we sought to rationally identify the ideal properties for small molecule ligands targeting FAP. Using a diverse set of FAPI derivatives with varying physicochemical properties and pharmacokinetics, the extent of target engagement as measured via NIR molecular imaging highlights design considerations for FAP targeting agents in autoimmune applications, whether for imaging or drug delivery. Ideal targeting ligand properties are context dependent and often depend on where antigen is expressed. For example, an extracellular matrix protein target likely has more stringent requirements for binding affinity than an internalizing cell surface receptor. For drug delivery applications, it may be advantageous to pair faster internalization with slower plasma clearance, assuming efficient lysosomal delivery following receptor endocytosis and continued cellular synthesis and trafficking of new receptor to the membrane. For imaging applications however, a rapidly clearing agent may provide optimal contrast at the cost of lower total delivery. As the interplay between internalization and plasma clearance is both complex and may be application-specific, a comprehensive net internalization analysis was performed in this work as a first step for probe optimization.

For FAPI, a moderate internalization half-life (*t*_*1*/2_ ~ 4–8 h) was measured in endogenous and overexpressing cells. The net internalization half-life depends on the target antigen and protein interaction and can vary from minutes to days^52^ and though it describes one step of receptor trafficking, it needs to be measured alongside surface expression to fully detail the kinetics. Transfected HEK293 cells overexpress FAP at ~1 million receptor per cell while the RAFLS cells were calculated to express only 25,000 receptors per cell (Table 2). Given the large difference in expression and relatively minor differences in internalization rate, there was significantly more targeted protein delivery per cell in the HEK293 internalization assay. Targeted delivery for HEK293-hFAP was much greater than the non-specific uptake. However, for lower-expressing RAFLS cells with reduced capacity for targeted delivery, the non-specific uptake constituted a significant fraction of the total internalized signal. The comparison of targeted and non-specific uptake in high/low expressing cells is salient for inflamed conditions, such as RA, where target expression can be relatively low and non-specific uptake increased. While low absolute expression of RAFLS may prove challenging to image and quantify in vivo, the moderately fast internalization rate was encouraging and FAPI ligands with slower plasma clearance rates were evaluated to assess potential benefits of prolonged FAP endocytosis over time on specific in vivo uptake.

First, the total uptake in diseased paws was compared for moderate and slow clearing 800CW probes. While the longer systemic half-lives for the ALB conjugate did improve the total MFI in diseased paws compared to probes without enhanced albumin binding, it also showed increased uptake in healthy paws, suggesting greater nonspecific or perfusion-driven signal. The impact of slower clearance on in vivo FAP binding was assessed via competition, where ALB conjugates were used to block the signal of the NIR imaging probe. Here, the absolute differences between blocked and unblocked groups were similar for animals given probes with/without albumin binding. We hypothesize the in vivo FAP expression in this model and/or internalization rates are not sufficient to differentiate between the imaging tracers tested. Though the slow clearing probes are unable to achieve higher signal to background ratio for imaging, they allow for in vivo monitoring of target engagement at longer times. While minimal improvements in contrast may limit the translatability of a slow-clearing FAPI ligand for molecular imaging tracer development, the impact of engineering the pharmacokinetics in the context of a therapeutic is less clear and will likely be application-dependent. While the results suggest that using FAPI ligands to deliver therapeutics/payload intracellularly in RA patients may be challenging based on a combination of slow trafficking and moderate in vivo expression, it demands careful consideration to payload potency. For example, peptide-based GLP-1R molecular imaging ligands—which exhibit similar binding affinity and pharmacokinetics as FAPI ligands—also show limited improvements in imaging contrast when engineered for slower plasma clearance^61^. However, prolonged target engagement for lipidated GLP-1R ligands using therapeutic peptides have seen success in the clinic^62^.

Although albumin-binding moieties provided limited benefits for FAP imaging in this work, alternative modifications in molecular structure that increased ligand hydrophilicity and pharmacokinetics improved target engagement. By conjugating a sulfonated Alexa Fluor 647 fluorophore to FAPI, the resulting probe showed significantly reduced membrane partitioning. While this may not be ideal for oral bioavailability, the probes evaluated in this work were dosed IV. For this parenteral route of administration, increased hydrophilicity and improved pharmacokinetics resulted in greater antigen binding, whereas lipophilic FAPI showed poor targeting from reduced plasma concentrations as a consequence of rapid clearance and large volume of distribution. Molecular imaging agents targeting transmembrane receptors are seldomly administered orally due to limited bioavailability, although some have shown promise in preclinical models^10^. While reduced gut absorption may be overcome by increasing dose for an inert fluorescent imaging agent, ideal tracers should effectively engage target to improve contrast, independent of route of administration. The results of this work suggest FAP targeted therapies—whether delivering a detectable label or pharmacologically active payload— may benefit with non-albumin binding modifications that increase overall hydrophilicity and pharmacokinetics.

## Methods

### Reagents

Unless otherwise specified all the reagents and solvents used in the synthesis of the various FAPI derivatives highlighted in this work were purchased from Millipore-Sigma (St. Louis, MO). Trifluoroacetic acid (TFA) was obtained from Chem-Impex, Wood Dale, IL. Alexa Fluor 647 (AF647) NHS ester was obtained from ThermoFisher (Waltham, MA). IRDye 800CW NHS ester was obtained from LI-COR Biosciences (Lincoln, NE). Fmoc-Glu-OtBu and 2,2-dimethyl-4-oxo-3,8,11-trioxa-5-azatridecan-13-oic acid were obtained from Combi-Blocks (San Diego, CA). ((3H-[1,2,3]triazolo[4,5-b]yrrolid-3-yl)oxy)tri(yrrolidine-1-yl)phosphonium hexafluorophosphate(V) (PyAOP) was obtained from Oakwood Chemicals (Estill, SC). Analytical LCMS and preparative HPLC methods are detailed in the Supporting Information. NMR spectra were collected using a Bruker Avance III HD spectrometer (Bruker, Billerica, MA).

### Protein binding equilibrium dialysis

CD1 mouse plasma protein binding was assessed via equilibrium dialysis. Dry membranes (HT Dialysis, HTD96b) were soaked in DI water for 15 min and then in 30% ethanol for another 15 min. After washing the membranes 3 times with DI water, the membranes were placed in dialysis buffer (potassium phosphate, 50 mM, pH 7.4) until the assay began. At the beginning of the assay, the membranes were placed in the dialysis block and 100 µL of buffer was added to both sides of the membrane to maintain membrane hydration/integrity. Each compound was diluted to 1 µM using plasma and then 100 µL of this mixture was added to one side of the membrane while 100 µl of plasma was placed in opposite well. The plate was then sealed and placed on an orbital shaker (150 rpm) in an incubator at 37 °C for 4 h. Afterwards, samples were taken from the donor and acceptor side of the well, vortexed, centrifuged at 3300 rpm for 15 min at 4 °C and then analyzed for compound content using LC/MS.

### Cell lines

HEK293 cells (ATCC, CRL-1573) were grown for 4 d in DMEM supplemented with 10% FBS containing 1% Pen/Strep. Cells were transferred to a 6-well plate and allowed to adhere before transfection. Master mixes were made using the Lipofectamine 3000 Transfection Reagent Kit and Opti-MEM-I. The pDNA and lipofectamine master mixes were combined at room temperature for 10 min and 250 µL of the DNA/Lipofectamine mixture was added dropwise to each well. Cells were selected using total growth media containing 0.6 µg/mL puromycin.

Cryopreserved human fibroblast-like synovial cells from rheumatoid arthritis patients were sourced from Cell Applications (HFLS-RA, Catalog 408RA-05a, San Diego, CA).

### Binding affinity and Cell Receptor Expression assays

Briefly, HEK293-hFAP cells were harvested with 0.05% trypsin, washed with PBS containing 0.1% BSA, and incubated in PBS w/ 0.1% BSA containing FAPI-AF647, FAPI-AF488 or FAPI-ALB-AF647 on ice. The concentration of each probe was varied from 0.78 nM – 200 nM to generate a complete binding curve. After 2 h, cells were centrifuged, washed twice with PBS w/ 0.1% BSA, and analyzed using a BD Fortessa flow cytometer (BD Biosciences, Woburn, MA).

To evaluate the binding affinity of unlabeled FAPI and FAPI-800CW compounds, a competition assay was developed to determine the relative binding affinity of unlabeled probes compared to FAPI-ALB-AF647 (*K*_*D*_ = 3.4 nM). Both the FAPI-ALB-AF647 and the unlabeled competitor were incubated at 60 nM each on HEK293-hFAP cells for 30 min on ice to approach steady-state binding. The competition assay cells were pelleted and washed twice with PBS w/ 0.1% BSA before being analyzed using a BD Fortessa flow cytometer. The loss in signal from the competition incubation was then compared to the signal of the FAPI-ALB-AF647 incubation alone to determine the relative binding affinity.

In addition to measuring cell surface affinity, the number of surface bound ligand was measured as an estimate of absolute cell surface FAP expression in HEK293-hFAP cells. Briefly, cells were labeled with 200 nM of FAPI-AF647, washed twice with PBS with 0.1% BSA, and analyzed using flow cytometer alongside MESF fluorescent beads (Bangs Laboratories, Fishers, IN). Binding data were plotted using GraphPad Prism and binding affinities were estimated using a one-site saturation model.

### Receptor internalization assay

The receptor internalization assay was adapted from a previously published method for peptides^61,63^. Briefly, HEK293-hFAP cells were sub-cultured into 6-well plates and allowed to adhere for 12–16 h. After washing cells with media, 100 nM of FAPI-AF488 in media was added to each well. Cells were incubated at 37 °C in the continuous presence of FAPI-AF488 and allowed to internalize. At each time point, plates were chilled, wells washed twice with PBS with 0.1% BSA, and cells lifted following Accutase incubation. Lastly, cells were resuspended in PBS or a dilution of 0.4% Trypan Blue before being analyzed via flow cytometry to differentiate between internal and surface fluorescence. The surface fluorescence was determined as (unquenched MFU – quenched MFU)/ɛ, where ɛ is the quenching efficiency determined by comparing the quenched and unquenched signals of cells that had been labeled briefly on ice to prevent internalization. The internal fluorescence was then calculated as total MFU – surface MFU. Non-specific uptake of small molecules was measured using cells where FAPI-AF488 was competed out with 100-fold excess concentration of the unlabeled FAPI. The corrected internal MFU values were plotted against the surface fluorescence integral; the slope of the linear curve is the internalization rate k_e_^64^. The same procedure was followed for the endogenous RAFLS cells.

HEK293-hFAP cells were seeded at ~2 × 10^5^ cells per well in a 8-well glass coverslip bottom dishes (Nunc). After attachment, cells were incubated at 37 °C with 200 nM FAPI reagent conjugated to Alexa-647 and images were immediately captured using Leica SP8 Confocal with live cell imaging capability from a time frame of 5 min to 24 h at 63X using oil objective. For colocalization into lysosomes, the cells were incubated overnight with 50 µg/mL of dextran AF488 10,000 MW (Invitrogen, D22910) and after washing cells were incubated at 37 °C with 200 nM FAPI-AF647. Time lapse images were captured using Operetta CLS High-Content Analysis System (PerkinElmer, Waltham, MA) from 5 min to 24 h with a 40X water-immersion objective.

### Animals

Collagen-induced-arthritis (CIA) was induced as described previously^54^. All animals were maintained at constant temperature and humidity under a 12 h light/dark cycle and fed with rodent chow (Lab Diet 5010 PharmaServ, Framingham, MA) and water ad libitum. Abbott / AbbVie is AAALAC (Association for Assessment and Accreditation of Laboratory Animal Care) accredited, and all procedures were approved by the AbbVie IACUC prior to initiation. Six- to eight-week-old male DBA/1 J mice were obtained from Jackson Labs (Bar Harbor, ME). Mice were immunized intradermally at the base of the tail with 100 µL of emulsion containing 100 µg of type II bovine collagen dissolved in 0.1 N acetic acid and 333 µg of heat-inactivated Mycobacterium tuberculosis H37Ra. Twenty-one days after immunization with collagen, mice were boosted intraperitoneally with 1 mg of Zymosan A (Sigma-Aldrich, St. Louis, MO), a nonspecific stimulus which serves to synchronize the onset of disease symptoms in this model^65^. Following the boost, mice were monitored daily for arthritis. Rear paws were evaluated for paw swelling using Dyer spring calipers. Mice were enrolled for the study beginning 3 days after the Zymosan boost at the first clinical signs of disease. The day marked by the clinical sign was set as ‘Day 0’ of disease progression. On day 7 of disease progression, the rear paw with the greater caliper score was selected for further analysis and considered the “enrolled” paw. At the termination of the experiment, animals were euthanized with Isoflurane and paws from each group were harvested and stored in 10% neutral buffered formalin for histopathology.

FAPI was dosed IV (51 nmol) via the tail vein to maintain concentrations above LLOQ for PK analysis while all other FAPI conjugates were dosed IV (1 or 2 nmol in 150 µL PBS) via the tail vein. To evaluate the plasma clearance, 10 µL blood samples were collected at 1, 3, 5, 15, 30, 60 and 180 min timepoints via retro-orbital blood draws. Whole blood was diluted with 10 mM EDTA in PBS and spun down to isolate plasma. Fluorescent ligand concentrations were quantified using a LI-COR Odyssey CLx scanner (Lincoln, NE) using a dilution series of fluorescent standards as previously reported^61^. Non-compartmental analysis was conducted using the concentration vs. time profiles for each probe with MATLAB. The predicted initial concentration, terminal phase half-life, exposure (AUC_0-inf_), AUC extrapolation percentage, volume of distribution at steady state, and terminal clearance were reported for each dosed probe.

### In vivo fluorescence imaging

Mice received intravenous injection of 1 nmol of FAPI conjugates (AF647 or 800CW). Fluorescence imaging was performed using the LI-COR Pearl imaging platform (Lincoln, NE) with an excitation/emission at 685 nm/720 nm and at 785 nm/820 nm to detect AF647 or 800CW fluorescence, respectively. Mean fluorescence intensity was determined using ImageStudio software (LI-COR, Lincoln, NE) by subtracting the background, drawing a region of interest around the paws, and using a measure function to determine the mean gray value.

### Ex vivo biodistribution

The following protocol was modified from previous protocols for quantifying uptake of NIR-labeled targeting ligands^66,67^. Briefly, after the last blood draw, mice were euthanized and the organs were resected, weighed, and snap frozen. The excised organs were incubated at 37 °C in a mixture of RIPA buffer in PBS supplemented with 6 mg/ml collagenase type IV for 30 min at 37 °C. After incubation, the organs were homogenized using a FB-120 Sonic Dismembrator. Subsequently, an equal volume of 0.25% trypsin/EDTA was added, and the solution was incubated for another 30 min at 37 °C before being homogenized using a FB-120 Sonic Dismembrator. Fluorescence intensity of final organ digests was measured using an Odyssey CLx (Li-Cor, Lincoln, NE). Identical digests were performed with FAPI-800CW to generate a calibration curve; bulk signal from organ digests was converted to absolute concentrations to quantify the %ID/g.

### Statistical analysis

Plot values are shown as mean +/- SEM. Data were analyzed for statistical significance using a multiple unpaired *t-*test with Welch correction using GraphPad Prism 10.

### Specific target blocking analysis

The competitive blocking of FAPI-800CW was utilized as a measure of in vivo target engagement between the hydrophilic probes (FAPI-AF647 or FAPI-ALB-647) and lipophilic probes (FAPI or FAPI-ALB). The signal from the healthy mouse no-block FAPI-800CW group was subtracted from each treatment group in the disease mouse model to approximate the FAPI specific signal in the treated groups at each timepoint. The maximum potential signal block was identified as the difference in signal between the disease no-block and the healthy no-block signal. The percent specific signal blocked for each probe was then calculated by taking the difference between the no-block disease signal and the blocking probe (FAPI-AF647 or FAPI for the non-ALB versions) signal over the maximum potential signal block and used to produce Fig. 7C. Similar analysis were performed for the half-life extended ALB variants.

### Histology

Formalin-fixed paraffin embedded sections of mouse CIA (knee) synovial tissue were sectioned at 5 µm. Immunohistochemistry was performed on the Leica Bond RX autostainer (Leica Biosystems, Deer Park, IL). Slides were baked and dewaxed on the Bond RX, followed by a heat-induced epitope retrieval step with Leica’s citrate buffer, Epitope Retrieval 1 (Catalog AR9961), for 30 min. Slides were blocked with both a Dual Endogenous Enzyme Block (Catalog S200389-2, Agilent, Santa Clara, CA) and Protein Block, Serum-Free (Catalog X090930-2, Agilent). The synovial samples were labeled with an anti-FAP antibody (Catalog ab207178, Abcam, Cambridge, UK) at a concentration of 2.5 µg/ml for 30 min followed by a hematoxylin counterstain. Slides were scanned with a Pannoramic 250 whole slide digital scanner (3DHISTECH Ltd, Budapest, Hungary).

## Supplementary information

Supplementary data

## Data Availability

The datasets used and/or analyzed during the current study are available from the corresponding author on reasonable request.
