# Supplementary material for: Increased imaging ligand hydrophilicity and improved pharmacokinetic properties provides enhanced in vivo targeting of fibroblast activation protein
Source: Npj Imaging. 2024 Aug 2;2:24. doi: 10.1038/s44303-024-00028-0 (PMC12118671; doi:10.1038/s44303-024-00028-0)
Supplement: Supplementary file 1 — Supplementary data [file 44303_2024_28_MOESM1_ESM.pdf]

SUPPLEMENTAL DATA

PROBE DESIGN

FAPI is a known high-affinity ligand for the tumor associated membrane serine protease fibroblast-activating protein (FAP) to which various imaging reagents have been conjugated[1]. The red dye (AF647) and near-IR dye (800CW) conjugates of both native FAPI and FAPI conjugated with a serum albumin binding small-molecule[2] were prepared as shown in figure 1. The original biphenyl acylsulfonamide albumin binding probe binds with relatively high affinity to domain III of human serum albumin and was utilized to keep the FAPI-fluorophore conjugate in serum to increase circulating half-life.

METHODS

Reagents: Unless otherwise specified all the reagents and solvents used were purchased from Millipore-Sigma, St. Louis MO. Trifluoroacetic acid (TFA) was obtained from Chem-Impex, Wood Dale, IL. 2-((1E,3E)-5-((E)-3,3-dimethyl-5-sulfonato-1-(3-sulfopropyl)indolin-2-ylidene)penta-1,3-dien-1-yl)-3-(6-((2,5-dioxocyclopentyl)oxy)-6-oxohexyl)-3-methyl-1-(3-sulfonatopropyl)-3H-indol-1-ium-5-sulfonate bis triethylammonium salt (**AF647-NHS**) was synthesized as described[3]. sodium 2-((E)-2-((E)-3-(2-((E)-3,3-dimethyl-5-sulfonato-1-(4-sulfonatobutyl)indolin-2-ylidene)ethylidene)-2-(4-sulfonatophenoxy)cyclohex-1-en-1-yl)vinyl)-1-(6-((2,5-dioxopyrrolidin-1-yl)oxy)-6-oxohexyl)-3,3-dimethyl-3H-indol-1-ium-5-sulfonate (**IR800CW-NHS**) was obtained from Licor Biosciences, Lincoln, NE. 4'-(trifluoromethyl)-[1,1'-biphenyl]-4-carboxylic acid, 4-cyanobenzenesulfonamide, (S)-4-((((9H-fluoren-9-yl)methoxy)carbonyl)amino)-5-(tert-butoxy)-5-oxopentanoic acid (Fmoc-Glu-OtBu) and 2,2-dimethyl-4-oxo-3,8,11-trioxa-5-azatridecan-13-oic acid were obtained from Combi-Blocks, San Diego, CA. ((3H-[1,2,3]triazolo[4,5-b]pyridin-3-yl)oxy)tri(pyrrolidin-1-yl)phosphonium hexafluorophosphate(V) (PyAOP) was obtained from Oakwood Chemicals, Estill, SC.

ABBREVIATIONS:

AA	ammonium acetate
AF647-NHS	2-((1E,3E)-5-((E)-3,3-dimethyl-5-sulfonato-1-(3-sulfopropyl)indolin-2-ylidene)penta-1,3-dien-1-yl)-3-(6-((2,5-dioxocyclopentyl)oxy)-6-oxohexyl)-3-methyl-1-(3-sulfonatopropyl)-3H-indol-1-ium-5-sulfonate bis triethylammonium salt. AlexaFluor™647-N-hydroxysuccinimide
APCI-MS	atmospheric pressure chemical ionization mass spectrometry
DMF	N,N-dimethylformamide
DMSO	dimethylsulfoxide
ESI-MS	electrospray ionization mass spectrometry
Fmoc-Glu-OtBu	(S)-4-((((9H-fluoren-9-yl)methoxy)carbonyl)amino)-5-(tert-butoxy)-5-oxopentanoic acid
HPLC	high-performance liquid chromatography
IR800CW-NHS	IR800CW™-N-hydroxysuccinimide
LCMS	liquid chromatography mass-spectrometry
MeCN	acetonitrile
PyAOP	((3H-[1,2,3]triazolo[4,5-b]pyridin-3-yl)oxy)tri(pyrrolidin-1-yl)phosphonium hexafluorophosphate(V)

RP-HPLC	reverse-phase high-performance liquid chromatography
R_t	Retention time
TFA	trifluoroacetic acid

SYNTHETIC METHODS

Analytical LCMS with TFA method 1, TFA method 2, AA method 1 and AA method 2: Analytical LCMS was performed on a Thermo MSQ-Plus mass spectrometer and Agilent 1100/1200 HPLC system running Xcalibur 2.0.7, Open-Access 1.4, and custom login software. The mass spectrometer was operated under positive APCI or ESI ionization conditions dependent on the system used, as noted in the text. The HPLC system comprised an Agilent Binary pump, degasser, column compartment, autosampler and diode-array detector, with a Polymer Labs ELS-2100 evaporative light-scattering detector. The column used was a Phenomenex Kinetex C8, 2.6 µm 100 Å (2.1mm × 30mm), at a temperature of 65°C. Elution times are reported as R_t.

"TFA method 1": A gradient of 5-100% acetonitrile (A) and 0.1% trifluoroacetic acid in water (B) was used, at a flow rate of 1.5 mL/min (0-0.05 min 5% A, 0.05-1.2 min 5-100% A, 1.2-1.4 min 100% A, 1.4-1.5 min 100-5% A. 0.25 min post-run delay). Elution times are reported as R_t.

"TFA method 2": A gradient of 5-100% acetonitrile (A) and 0.1% trifluoroacetic acid in water (B) was used, at a flow rate of 1.5 mL/min (0-0.1 min 5% A, 0.1-5.2 min 5-100% A, 5.2-5.7 min 100% A, 5.7-6.0 min 100-5% A. 0.25 min post-run delay). Elution times are reported as R_t.

"AA method 1": A gradient of 5-100% acetonitrile (A) and 10 mM ammonium acetate in water (B) was used, at a flow rate of 1.5 mL/min (0-0.05 min 5% A, 0.05-1.2 min 5-100% A, 1.2-1.4 min 100% A, 1.4-1.5 min 100-5% A. 0.25 min post-run delay). Elution times are reported as R_t.

"AA method 2": A gradient of 5-100% acetonitrile (A) and 10 mM ammonium acetate in water (B) was used, at a flow rate of 1.5 mL/min (0-0.1 min 5% A, 0.1-5.2 min 5-100% A, 5.2-5.7 min 100% A, 5.7-6.0 min 100-5% A. 0.25 min post-run delay). Elution times are reported as R_t.

Analytical LCMS with AA method 3 and FA method 1: Analytical LCMS was performed on a Thermo MSQ-Plus mass spectrometer and Agilent 1100/1200 HPLC system running Xcalibur 2.0.7, Open-Access 1.4, and custom login software. The HPLC system comprised an Agilent Binary pump, degasser, column compartment, autosampler and diode-array detector, with a Polymer Labs ELS-2100 evaporative light-scattering detector. The column used was a Waters Cortecs C18+, 2.7 µm 100Å (2.1mm × 30mm), at a temperature of 40°C. Elution times are reported as R_t.

"AA method 3": A gradient of 5-100% acetonitrile (A) and 10 mM ammonium acetate water:acetonitrile (98:2) (B) was used, at a flow rate of 1.5 mL/min (0-0.05 min 0% A, 0.05-2.8 min 0-100% A, 2.8-3.0 min 100-0% A, 160-1500 amu positive/negative ESI-MS ionization. Elution times are reported as R_t.

"FA method 1": A gradient of 5-100% acetonitrile (A) and 0.1% formic acid water:acetonitrile (98:2) (B) was used, at a flow rate of 1.5 mL/min (0-0.05 min 0% A, 0.05-2.8 min 0-100% A, 2.8-3.0 min 100-0% A, 160-1500 amu positive/negative ESI-MS ionization. Elution times are reported as R_t.

Preparative HPLC TFA method 1. RP-HPLC was performed on a Gilson HPLC system equipped with liquid handler (Gilson-215) and UV/Vis detector using a Waters Deltapak C18

column (5 µm, 100 Å, 200 x 21.2 mm) eluted with A (0.1% TFA-water):B (acetonitrile) [0-5 min: 5% A; 5-35 min linear gradient to 100% B, 3.16%/min linear gradient] with a 20 mL/min flowrate.

Preparative HPLC TFA method 2. RP-HPLC was performed on a Gilson HPLC system equipped with liquid handler (Gilson-215) and UV/Vis detector using a Phenomenex Gemini NX-C18 column (5 µm, 110 Å, 250 x 25 mm) eluted with A (0.1% TFA-water):B (acetonitrile) [0-5 min: 2% A; 5-35 min linear gradient to 100% B, 3.16%/min linear gradient] with a 20 mL/min flowrate.

NMR Spectra were collected in DMSO-d_6 at room temperature (27 °C) on a Bruker Avance III HD spectrometer equipped with a TCI cryoprobe. ^{1}H spectra were acquired with SW= 16 ppm, 3.65 s acquisition time & 8-24 scans. ^{13}C spectra were acquired with SW 200 ppm, 0.205 s acquisition time & 2 scans.

(S)-N-(2-(2-cyano-4,4-difluoropyrrolidin-1-yl)-2-oxoethyl)-6-(3-(piperazin-1-yl)propoxy)quinoline-4-carboxamide bis-2,2,2-trifluoroacetate (FAPI):

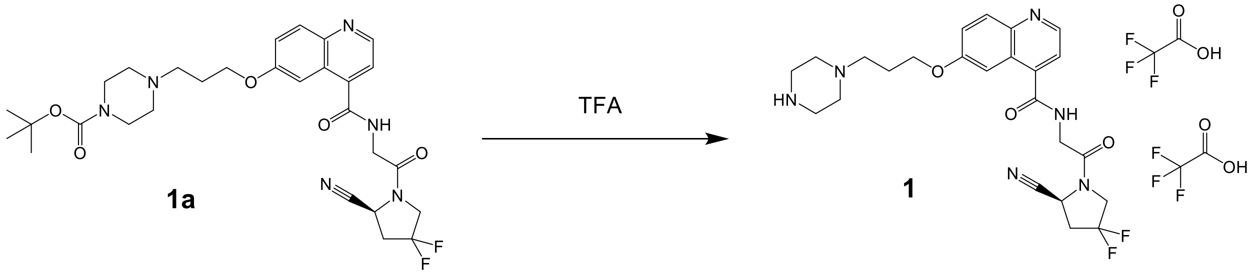

tert-butyl (S)-4-(3-((4-((2-(2-cyano-4,4-difluoropyrrolidin-1-yl)-2-oxoethyl)carbamoyl)quinolin-6-yl)oxy)propyl)piperazine-1-carboxylate (FAPI-BOC, 1a) synthesized by Enamine (Kyiv, Ukraine) via the route described[1] (50 mg, 0.085 mmol) was treated with neat TFA (1 mL) and immediately evaporated to dryness under a stream of dry nitrogen gas to give **1** (51.4 mg, 84.4%) as a colorless glass LCMS TFA method 1: R_t = 0.45 min, ESI-MS *m/z* 487.1$(M+H)^+$; LCMS AA method 3: R_t = 0.925 min, ESI-MS *m/z* 487.2 $(M+H)^+$, 485.2 $(M-H)^-$; ^{1}H NMR (499 MHz, DMSO) δ_H 9.12 (t, *J* = 6.0 Hz, 1H), 9.06 (s, 2H), 8.86 (d, *J* = 4.3 Hz, 1H), 8.03 (d, *J* = 9.2 Hz, 1H), 7.91 (d, *J* = 2.8 Hz, 1H), 7.57 (d, *J* = 4.4 Hz, 1H), 7.49 (dd, *J* = 9.2, 2.8 Hz, 1H), 5.16 (dd, *J* = 9.4, 2.9 Hz, 1H), 4.35 (m, *J* = 11.7, 4.7 Hz, 1H), 4.22 – 4.11 (m, 2H), 3.44 – 3.09 (m, 6H), 3.03 – 2.79 (m, 2H), 2.18 (p, *J* = 6.3 Hz, 2H); ^{13}C NMR (125 MHz, DMSO-d_6) δ_C 168.03, 167.34, 156.66, 147.53, 143.9, 140.77, 130.62, 126.73, 125.37, 122.47, 119.33, 117.75, 104.64, 65.27, 53.51 (2 C), 51.24, 44.06, 41.49, 41.36, 50.82 (2 C), 36.22, 23.92.

3-(6-(4-(3-((4-((2-((S)-2-cyano-4,4-difluoropyrrolidin-1-yl)-2-oxoethyl)carbamoyl)quinolin-6-yl)oxy)propyl)piperazin-1-yl)-6-oxohexyl)-2-((1E,3E)-5-((E)-3,3-dimethyl-5-sulfo-1-(3-sulfopropyl)indolin-2-ylidene)penta-1,3-dien-1-yl)-3-methyl-1-(3-sulfopropyl)-3H-indol-1-ium-5-sulfonate 2,2,2-trifluoroacetate (FAPI-AF647):

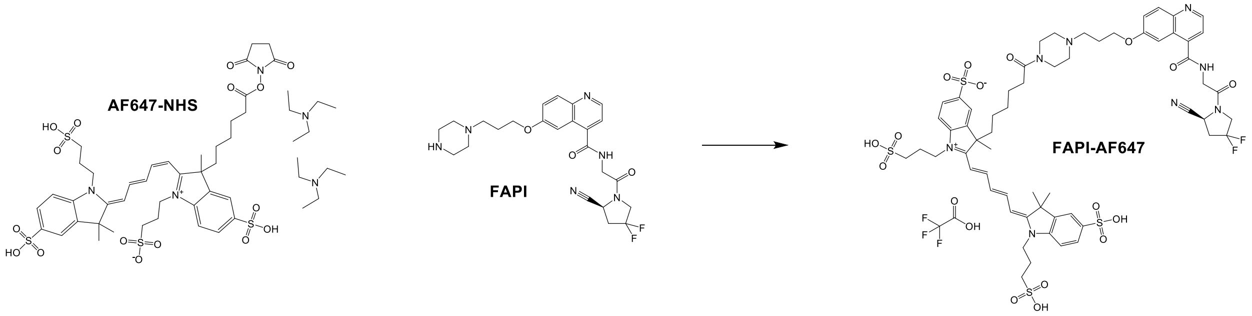

2-((1E,3E)-5-((E)-3,3-dimethyl-5-sulfonato-1-(3-sulfopropyl)indolin-2-ylidene)penta-1,3-dien-1-yl)-3-(6-((2,5-dioxocyclopentyl)oxy)-6-oxohexyl)-3-methyl-1-(3-sulfonatopropyl)-3H-indol-1-ium-5-sulfonate bis triethylammonium salt (**AF647-NHS**, 24.32 mg, 0.021 mmol) was combined with (S)-N-(2-(2-cyano-4,4-difluoropyrrolidin-1-yl)-2-oxoethyl)-6-(3-(piperazin-1-yl)propoxy)quinoline-4-carboxamide bis-2,2,2-trifluoroacetate (**FAPI**, 10 mg, 0.014 mmol) in 1 mL anhydrous DMSO containing 2% DIEA (v/v) and shaken at ambient overnight. The crude reaction mixture was diluted to 3 mL with 90% DMSO/water (v/v) and purified by preparative HPLC Preparative HPLC TFA method 1. Fractions containing the desired product were combined and lyophilized to give **FAPI-AF647** as a dark blue solid (7.6 mg, 37.7%): LCMS TFA method 2: R_t = 1.60 min, ESI-MS *m/z* 664.1 $(M+2H)^{2+}$; LCMS AA method 2: R_t = 1.55 min, ESI-MS *m/z* 664.1 $(M+2H)^{2+}$, 1327.6 $(M+H)^{+}$; LCMS FA method 1: R_t = 0.97 min, ESI-MS *m/z* 664.2 $(M+2H)^{2+}$, 662.2 $(M-H)^{-}$; LCMS AA method 3: R_t = 0.851 min, ESI-MS *m/z* 664.2 $(M+2H)^{2+}$, 662.0 $(M-H)^{-}$, 1325.4 $(M-2H)^{2-}$.

4-((E)-2-((E)-2-(3-((E)-2-(1-(6-(4-(3-((4-((2-((S)-2-cyano-4,4-difluoropyrrolidin-1-yl)-2-oxoethyl)carbamoyl)quinolin-6-yl)oxy)propyl)piperazin-1-yl)-6-oxohexyl)-3,3-dimethyl-5-sulfo-3H-indol-1-ium-2-yl)vinyl)-2-(4-sulfophenoxy)cyclohex-2-en-1-ylidene)ethylidene)-3,3-dimethyl-5-sulfoindolin-1-yl)butane-1-sulfonate 2,2,2-trifluoroacetate (FAPI-800CW):

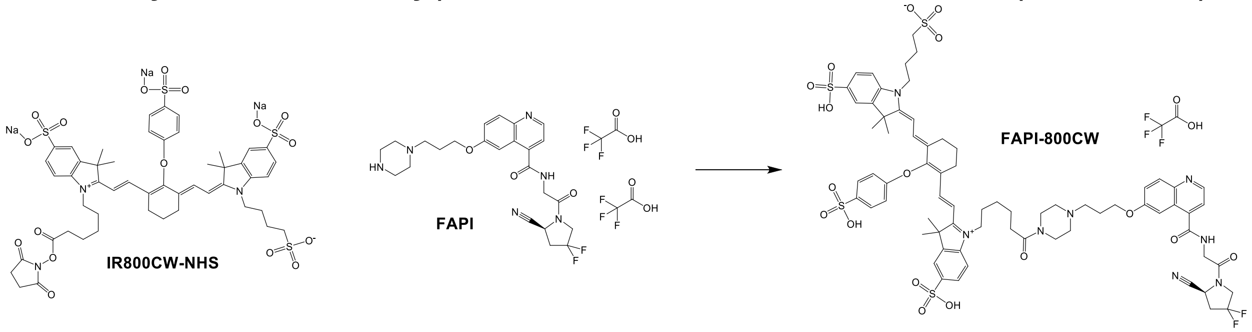

sodium 2-((E)-2-((E)-3-(2-((E)-3,3-dimethyl-5-sulfonato-1-(4-sulfonatobutyl)indolin-2-ylidene)ethylidene)-2-(4-sulfonatophenoxy)cyclohex-1-en-1-yl)vinyl)-1-(6-((2,5-dioxopyrrolidin-1-yl)oxy)-6-oxohexyl)-3,3-dimethyl-3H-indol-1-ium-5-sulfonate (**IR800CW-NHS**, 15 mg, 0.013 mmol) was combined with (S)-N-(2-(2-cyano-4,4-difluoropyrrolidin-1-yl)-2-oxoethyl)-6-(3-(piperazin-1-yl)propoxy)quinoline-4-carboxamide bis-2,2,2-trifluoroacetate (**FAPI**, 10 mg, 0.014 mmol) in 1 mL anhydrous DMSO containing 2% DIEA (v/v) and shaken at ambient overnight. The crude reaction mixture was diluted to 3 mL with 90% DMSO/water (v/v) and purified by preparative HPLC Preparative HPLC TFA method 2. Fractions containing the desired product were combined and lyophilized to give **FAPI-AF647** as a green solid (8.5 mg, 41.7%): LCMS TFA method 2: R_t = 2.01 min, ESI-MS *m/z* 744.4 $(M+H+Na)^{2+}$; LCMS AA method 1: R_t = 0.55 min, ESI-MS *m/z* 1472.31 $(M+H)^{+}$, 1327.6 $(M+H)^{+}$; LCMS FA method 1: R_t = 0.915 min, ESI-MS *m/z* 734.2 $(M-2H)^{2-}$, 732.8 $(M-H)^{-}$; LCMS AA method 3: R_t = 1.016 min, ESI-MS *m/z* 736.2 $(M+2H)^{2+}$, 734.2 $(M-2H)^{2-}$, 1325.4 $(M-2H)^{2-}$.

N-((4-cyanophenyl)sulfonyl)-4'-(trifluoromethyl)-[1,1'-biphenyl]-4-carboxamide (1):

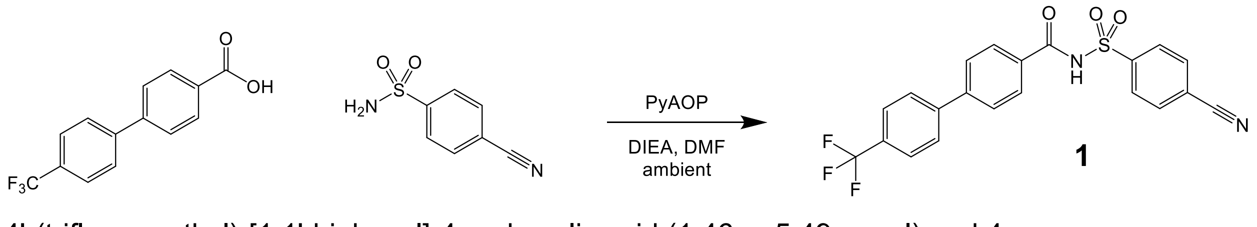

4'-(trifluoromethyl)-[1,1'-biphenyl]-4-carboxylic acid (1.46 g, 5.49 mmol) and 4-cyanobenzenesulfonamide (1.00 g, 5.49 mmol) were combined in 15 mL anhydrous DMF.

When fully dissolved, PyAOP (3.57 g, 6.86 mmol) was added and when fully dissolved, DIEA (2.13 g, 16.5 mmol) was added and the reaction shaken at ambient for overnight. The reaction was diluted with 90% DMSO/water and purified by Preparative HPLC TFA method 1 go give the desired product **1** (1.22 g, 51.7 %): LCMS FA method 1: R_t = 1.82 min, ESI-MS *m/z* 431.0 $(M+H)^+$, 428.8 $(M-H)^-$; LCMS AA method 3: R_t = 1.32 min, ESI-MS *m/z* 431.0 $(M+H)^+$; 429.0 $(M-H)^-$; ^1H NMR (499 MHz, DMSO-d_6) δ_H 8.22 – 8.17 (m, 2H), 8.17 – 8.12 (m, 2H), 8.04 – 8.00 (m, 2H), 8.00 – 7.95 (m, 2H), 7.92 – 7.83 (m, 4H); ^{13}C NMR (125 MHz, DMSO-d_6) δ_C 165.5, 143.77, 142.91 (2 C), 133.21 (2 C), 131.26, 129.29 (2 C), 128.59, 128.32 (2 C), 127.8 (2 C), 127.12 (2 C), 125.82 (2 C), 124.16, 117.54, 115.72.

N-((4-(aminomethyl)phenyl)sulfonyl)-4'-(trifluoromethyl)-[1,1'-biphenyl]-4-carboxamide (2):

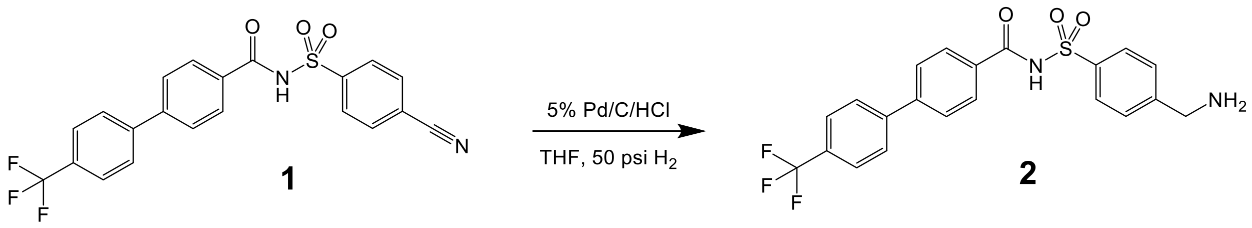

N-((4-cyanophenyl)sulfonyl)-4'-(trifluoromethyl)-[1,1'-biphenyl]-4-carboxamide (**1**, 1.047 g, 2.43 mmol) in THF (20 mL) and 4 M HCl in dioxane (1.216 mL, 4.87 mmol) in a Barnstead Hast C reactor was reduced with 5% Pd/C (wet JM#9) (0.67 g, 2.8 mmol) was reduced with 50 psi hydrogen at 25 C. The mixture was filtered through a polypropylene membrane and the solids washed with THF to give the desired product 2 (1.30 g, 92.3%) which was used without further purification. A sample (20 mg) was dissolved in 90% DMSO/water (v/v, 1.5 mL) and purified by Preparative HPLC TFA method 1 to give the desired product **2** as the 2,2,2-trifluoroacetic acid salt: LCMS FA method 1: R_t = 1.22 min, ESI-MS *m/z* 435.0 $(M+H)^+$, 869.2 $(2M+H)^+$, 432.8 $(M-H)^-$; LCMS AA method 3: R_t = 1.21 min, ESI-MS *m/z* 435.2 $(M+H)^+$, 892.0 $(2M+Na)^+$, 433.0 $(M-H)^-$, 889.0 $(2M-2H+Na)^+$.

tert-butyl (2-(2-(2-oxo-2-((4-(N-(4'-(trifluoromethyl)-[1,1'-biphenyl]-4-carbonyl)sulfamoyl)benzyl)-amino)ethoxy)ethoxy)ethyl)carbamate (3):

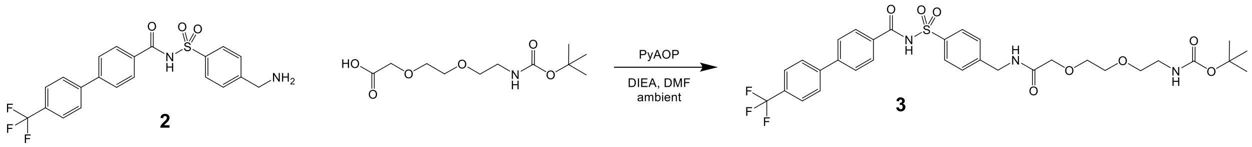

To N-((4-(aminomethyl)phenyl)sulfonyl)-4'-(trifluoromethyl)-[1,1'-biphenyl]-4-carboxamide (**2**, 106.4 mg, 0.245 mmol) and 2,2-dimethyl-4-oxo-3,8,11-trioxa-5-azatridecan-13-oic acid (77 mg, 0.294 mmol) were dissolved in 1 mL anhydrous DMF was added PyAOP (153 mg, 0.294 mmol), then DIEA (95 mg, 0.735 mmol). The reaction was shaken at ambient for 1h. The reaction was diluted to 3 mL with 90% DMSO/water (v/v) and purified by Preparative HPLC TFA method 2 to give the desired product **3**: LCMS FA method 1: R_t = 1.98 min, ESI-MS *m/z* 580.0 $(M+H-Boc)^+$; 678.0 $(M-H)^-$.

N-((4-((2-(2-(2-aminoethoxy)ethoxy)acetamido)methyl)phenyl)sulfonyl)-4'-(trifluoromethyl)-[1,1'-biphenyl]-4-carboxamide 2,2,2-trifluoroacetate (4):

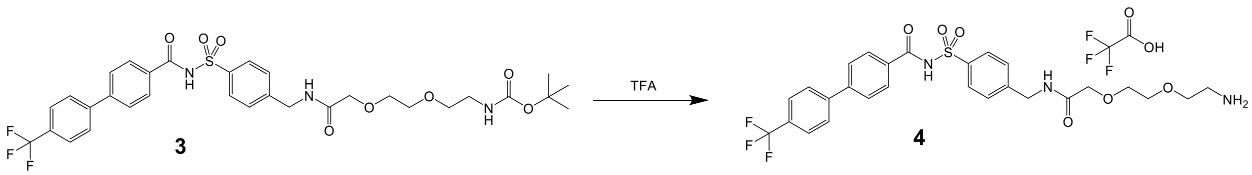

tert-butyl (2-(2-(2-oxo-2-((4-(N-(4'-(trifluoromethyl)-[1,1'-biphenyl]-4-carbonyl)sulfamoyl)benzyl)-amino)ethoxy)ethoxy)ethyl)carbamate (**3**, 139.5 mg, 0.205 mmol) was dissolved in 1 mL TFA and immediately evaporated to dryness under a stream of dry nitrogen gas to give the desired product **4** (142 mg, 100%) which was used without further purification: LCMS FA method 1: R_t = 1.37 min, ESI-MS *m/z* 579.8 $(M+H)^+$; 578.0 $(M-H)^-$.

tert-butyl (S)-15-((((9H-fluoren-9-yl)methoxy)carbonyl)amino)-3,12-dioxo-1-(4-(N-(4'-(trifluoro-methyl)-[1,1'-biphenyl]-4-carbonyl)sulfamoyl)phenyl)-5,8-dioxa-2,11-diazahexadecan-16-oate (5):

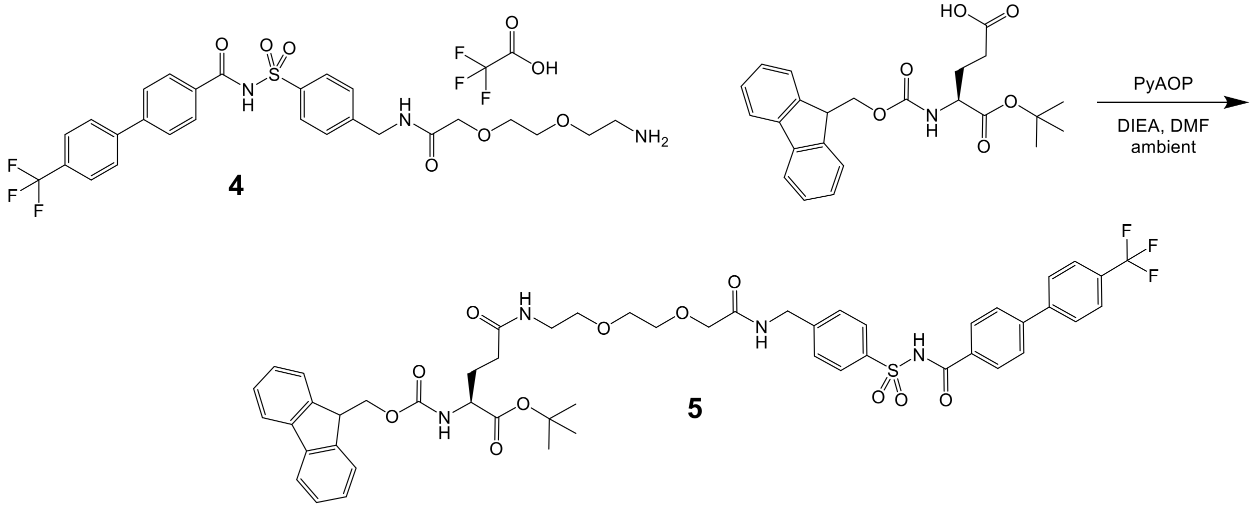

To N-((4-((2-(2-(2-aminoethoxy)ethoxy)acetamido)methyl)phenyl)sulfonyl)-4'-(trifluoromethyl)-[1,1'-biphenyl]-4-carboxamide 2,2,2-trifluoroacetate (**4**, 142 mg, 0.205 mmol) and Fmoc-Glu-OtBu (105 mg, 0.246 mmol) dissolved in 2 mL anhydrous DMF was added PyAOP (128 mg, 0.246 mmol) and DIEA (148 mg, 1.145 mmol). The reaction was shaken at ambient for 1 h. The reaction was diluted to 4 mL with 90% DMSO/water (v/v) and purified by Preparative HPLC TFA method 2 to give the desired product **5** (182.7 mg, 90.4%): LCMS FA method 1: R_t = 2.39 min, failed to show ions in ESI-MS.

(S)-15-((((9H-fluoren-9-yl)methoxy)carbonyl)amino)-3,12-dioxo-1-(4-(N-(4'-(trifluoromethyl)-[1,1'-biphenyl]-4-carbonyl)sulfamoyl)phenyl)-5,8-dioxa-2,11-diazahexadecan-16-oic acid (6):

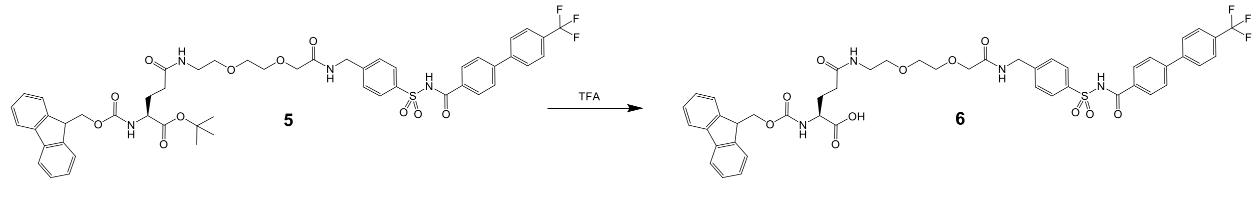

tert-butyl (S)-15-((((9H-fluoren-9-yl)methoxy)carbonyl)amino)-3,12-dioxo-1-(4-(N-(4'-(trifluoro-methyl)-[1,1'-biphenyl]-4-carbonyl)sulfamoyl)phenyl)-5,8-dioxa-2,11-diazahexadecan-16-oate (**5**, 182.7 mg, 0.185 mmol) was dissolved in 1 mL TFA and immediately evaporated to dryness under a stream of dry nitrogen gas. The TFA treatment was repeated to give the desired

product **6** (172 mg, 100%) which was used without further purification: LCMS FA method 1: R_t = 1.89 min, ESI-MS *m/z* 931.2 $(M+H)^+$; 929.0 $(M-H)^-$; LCMS AA method 3: R_t = 1.31 min, ESI-MS *m/z* 931.4 $(M+H)^+$; 929.0 $(M-H)^-$.

(9H-fluoren-9-yl)methyl ((S)-16-(4-(3-((4-((2-((S)-2-cyano-4,4-difluoropyrrolidin-1-yl)-2-oxoethyl)carbamoyl)quinolin-6-yl)oxy)propyl)piperazin-1-yl)-3,12,16-trioxo-1-(4-(N-(4'-(trifluoromethyl)-[1,1'-biphenyl]-4-carbonyl)sulfamoyl)phenyl)-5,8-dioxa-2,11-diazahexadecan-15-yl)carbamate 2,2,2-trifluoroacetate (7):

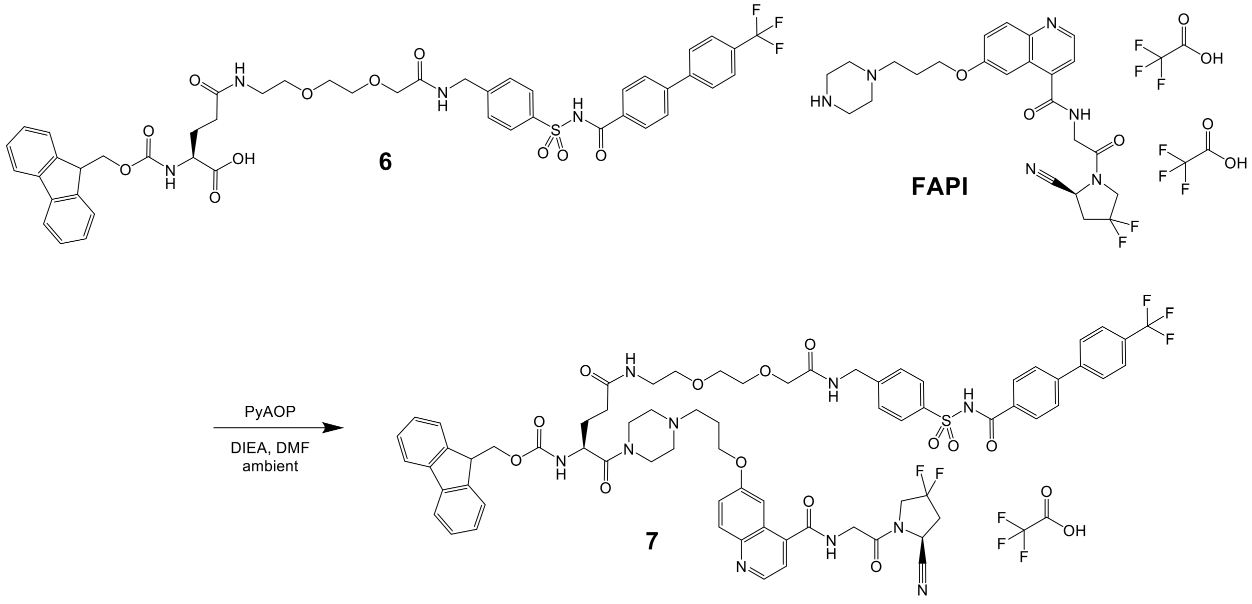

To (S)-15-((((9H-fluoren-9-yl)methoxy)carbonyl)amino)-3,12-dioxo-1-(4-(N-(4'-(trifluoromethyl)-[1,1'-biphenyl]-4-carbonyl)sulfamoyl)phenyl)-5,8-dioxa-2,11-diazahexadecan-16-oic acid (**6**, 43.8 mg, 0.047 mmol) and **FAPI** (33.6 mg, 0.047 mmol) in 1 mL anhydrous DMF was added PyAOP (29.4 mg, 0.056 mmol) and DIEA (30.4 mg, 0.235 mmol). The reaction was shaken at ambient for 1 h. The reaction was diluted to 3 mL with 90% DMSO/water (v/v) and purified by Preparative HPLC TFA method 2 to give the desired product **7** (46.2 mg, 64.9%): LCMS FA method 1: R_t = 1.76 min, ESI-MS *m/z* 700.2 $(M+2H)^{2+}$.

6-(3-(4-((S)-15-amino-3,12-dioxo-1-(4-(N-(4'-(trifluoromethyl)-[1,1'-biphenyl]-4-carbonyl)sulfamoyl)phenyl)-5,8-dioxa-2,11-diazahexadecan-16-oyl)piperazin-1-yl)propoxy)-N-(2-((S)-2-cyano-4,4-difluoropyrrolidin-1-yl)-2-oxoethyl)quinoline-4-carboxamide bis-2,2,2-trifluoroacetate (FAPI-albumin):

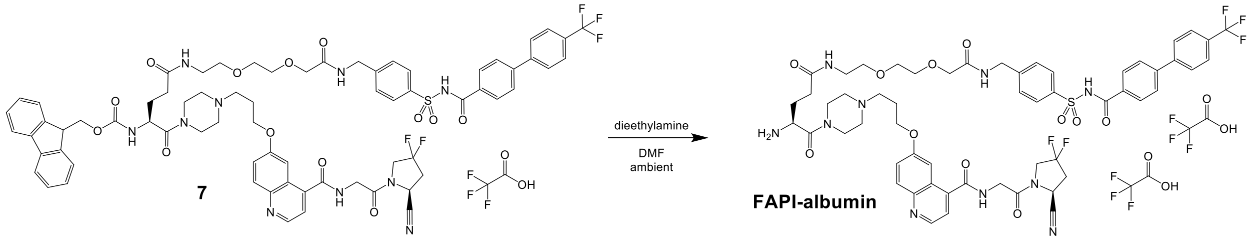

(9H-fluoren-9-yl)methyl ((S)-16-(4-(3-((4-((2-((S)-2-cyano-4,4-difluoropyrrolidin-1-yl)-2-oxoethyl)carbamoyl)quinolin-6-yl)oxy)propyl)piperazin-1-yl)-3,12,16-trioxo-1-(4-(N-(4'-(trifluoromethyl)-[1,1'-biphenyl]-4-carbonyl)sulfamoyl)phenyl)-5,8-dioxa-2,11-diazahexadecan-15-yl)carbamate 2,2,2-trifluoroacetate (**7**, 46.2 mg, 0.031 mmol) was dissolved in 1 mL 1:4 diethylamine:anhydrous DMF and shaken at ambient for 15 min. The solvent was partially

removed under a stream of dry nitrogen gas. The residue was diluted to 3 mL with 90% DMSO/water (v/v) and purified by Preparative HPLC TFA method 2 to give the desired product **FAPI-albumin** (also referred to as **FAPI-ALB** in this manuscript) (41.2 mg, 96.0%): LCMS FA method 1: R_t = 1.13 min, ESI-MS *m/z* 393.2 $(M+3H)^{3+}$, 589.4 $(M+2H)^{2+}$, 1177.6 $(M+H)^{+}$, 1175.0 $(M-H)^{-}$; LCMS AA method 3: R_t = 1.38 min, ESI-MS *m/z* 589.2 $(M+2H)^{2+}$, 1177.6 $(M+H)^{+}$, 587.2 $(M-2H)^{2-}$, 1175.4 $(M-H)^{-}$.

3-((S)-15-(4-(3-((4-((2-((S)-2-cyano-4,4-difluoropyrrolidin-1-yl)-2-oxoethyl)carbamoyl)quinolin-6-yl)oxy)propyl)piperazine-1-carbonyl)-3,12,17-trioxo-1-(4-(N-(4'-(trifluoromethyl)-[1,1'-biphenyl]-4-carbonyl)sulfamoyl)phenyl)-5,8-dioxa-2,11,16-triazadocosan-22-yl)-2-((1E,3E)-5-((E)-3,3-dimethyl-5-sulfo-1-(3-sulfopropyl)indolin-2-ylidene)penta-1,3-dien-1-yl)-3-methyl-1-(3-sulfopropyl)-3H-indol-1-ium-5-sulfonate 2,2,2-trifluoroacetate (FAPI-albumin-AF647):

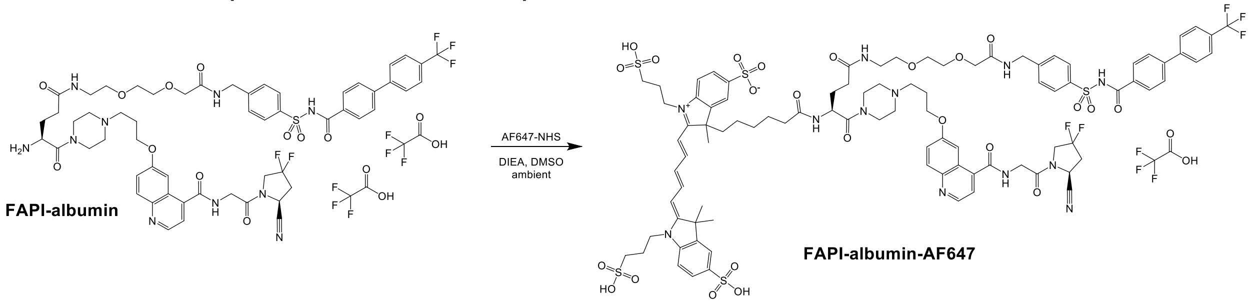

FAPI-albumin (13.5 mg, 0.0096 mmol) and **AF647-NHS** (16 mg, 0.014 mmol) were combined in 1 mL anhydrous DMSO containing 2% DIEA (v/v) and shaken at ambient overnight. The reaction was diluted to 3 mL with 90% DMSO/water (v/v) and purified by Preparative HPLC TFA method 2 to give the desired product **FAPI-albumin-AF647** (12.2 mg, 59.6%): LCMS FA method 1: R_t = 1.55 min, ESI-MS *m/z* 673.4 $(M+3H)^{3+}$, 1009.2 $(M+2H)^{2+}$, 671.8 $(M-3H)^{3-}$, 1008.4 $(M-2H)^{2-}$; LCMS AA method 3: R_t = 1.07 min, ESI-MS *m/z* 1009.6 $(M+2H)^{2+}$, 671.2 $(M-3H)^{3-}$, 1007.6 $(M-2H)^{2-}$.

4-((E)-2-((E)-2-(3-((E)-2-(1-((S)-15-(4-(3-((4-((2-((S)-2-cyano-4,4-difluoropyrrolidin-1-yl)-2-oxoethyl)carbamoyl)quinolin-6-yl)oxy)propyl)piperazine-1-carbonyl)-3,12,17-trioxo-1-(4-(N-(4'-(trifluoromethyl)-[1,1'-biphenyl]-4-carbonyl)sulfamoyl)phenyl)-5,8-dioxa-2,11,16-triazadocosan-22-yl)-3,3-dimethyl-5-sulfo-3H-indol-1-ium-2-yl)vinyl)-2-(4-sulfophenoxy)cyclohex-2-en-1-ylidene)ethylidene)-3,3-dimethyl-5-sulfoindolin-1-yl)butane-1-sulfonate 2,2,2-trifluoroacetate (FAPI-albumin-800CW):

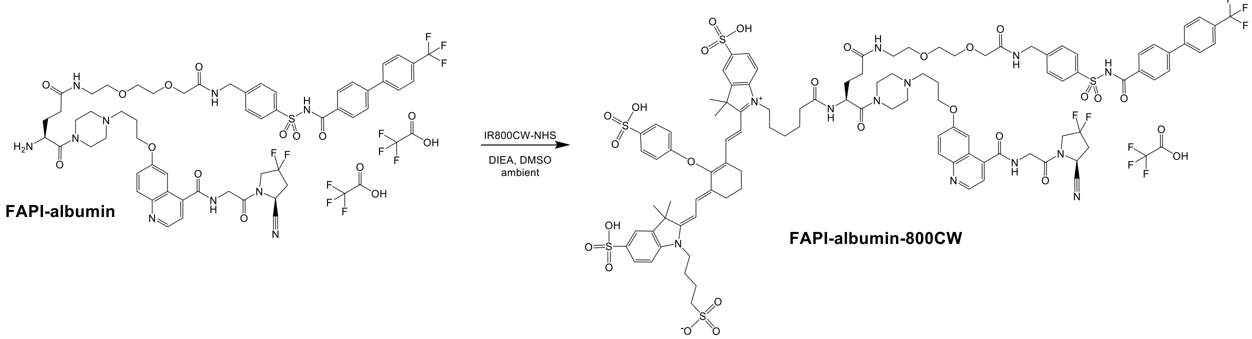

FAPI-albumin (15 mg, 0.0107 mmol) and **IR800CW-NHS** (13.7 mg, 0.012 mmol) were combined in 1 mL anhydrous DMSO containing 2% DIEA (v/v) and shaken at ambient overnight. The reaction was diluted to 3 mL with 90% DMSO/water (v/v) and purified by Preparative HPLC TFA method 2 to give the desired product **FAPI-albumin-AF647** (12.2 mg, 59.6%): LCMS FA method 1: R_t = 1.58 min, ESI-MS *m/z* 721.0 $(M+3H)^{3+}$, 1081.2 $(M+2H)^{2+}$,

718.9 $(M-3H)^{3-}$, 1079.6 $(M-2H)^{2-}$; LCMS AA method 3: R_t = 1.14 min, ESI-MS *m/z* $(M+3H)^{3+}$, 1083.0 $(M+2H)^{2+}$, 539.0 $(M-4H)^{4-}$, 719.4 $(M-3H)^{3-}$, 1078.6 $(M-2H)^{2-}$.

SUPPLEMENTAL FIGURES

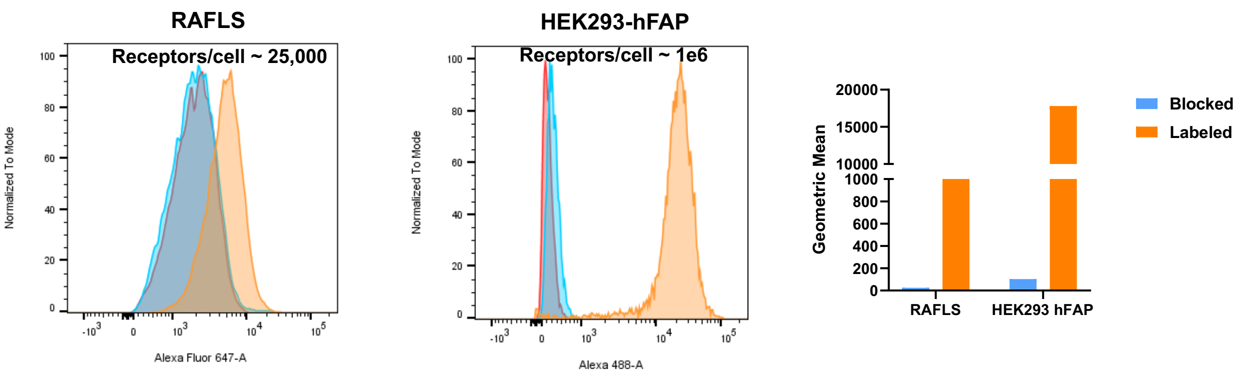

Figure S1. Specific binding of FAPI to both overexpressing and endogenous cells.

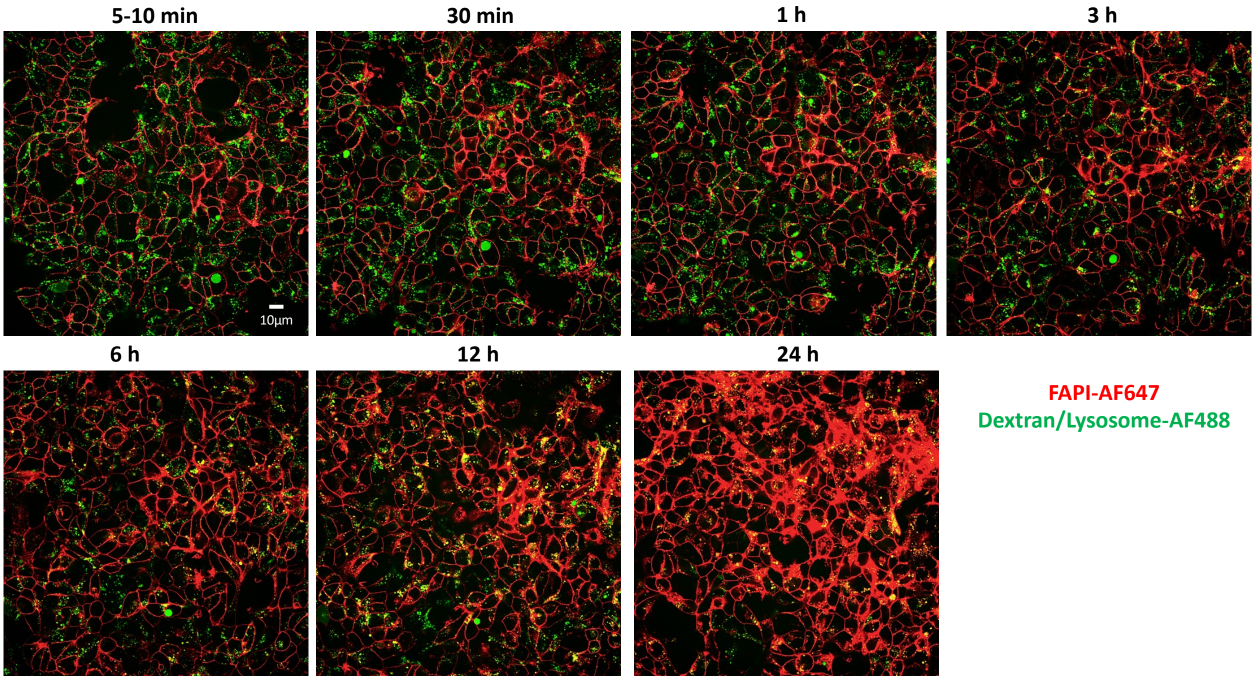

Figure S2. Time-lapse cell imaging of HEK293-hFAP cells labeled with FAPI-AF647 (red) which co-localizes with lysosomes (green) visualized by yellow punctures.

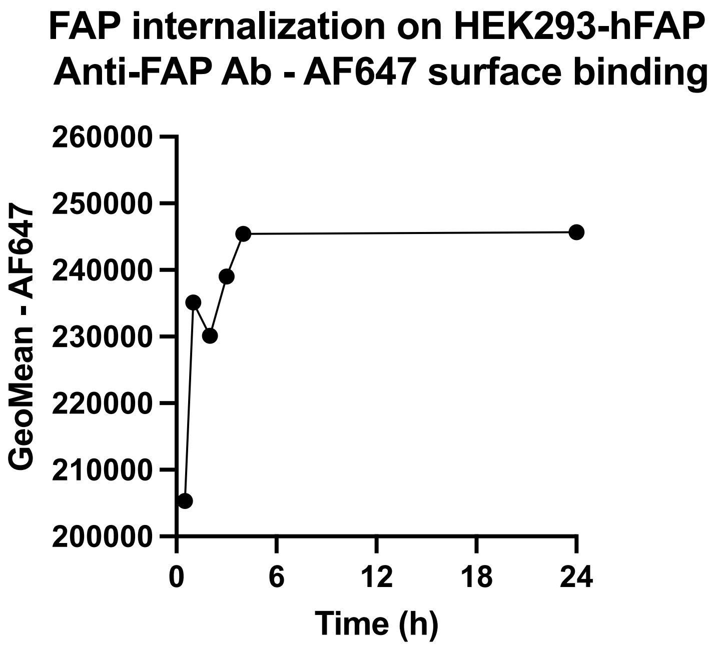

Figure S3. Constant surface expression of FAP on HEK293 overexpressing cells over time.

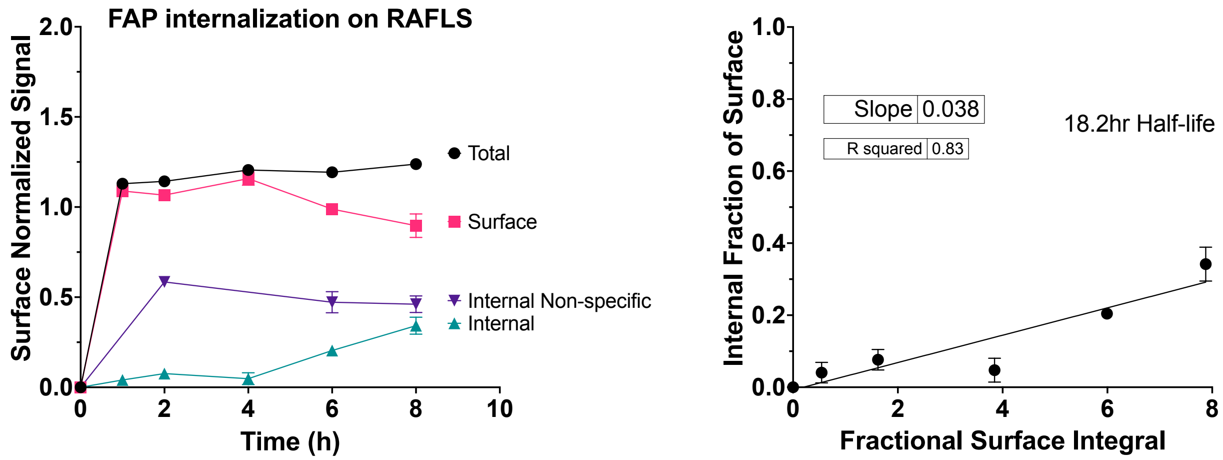

Figure S4. RAFLS internalization kinetics from a donor with higher non-specific uptake relative to that observed for a different donor shown in Figure 3.

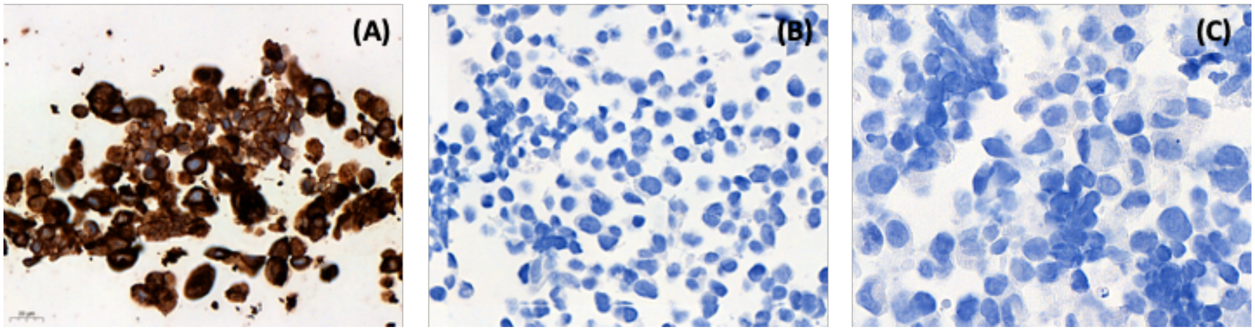

Figure S5. (A) Representative image of immunostaining with the anti-FAP IHC antibody in FAP-overexpressing HEK293 cells; (B) Lack of immunostaining with the anti-FAP antibody in FAP-negative HEK293 cells and (C) IHC with an isotype control antibody in FAP-overexpressing HEK293 cells.

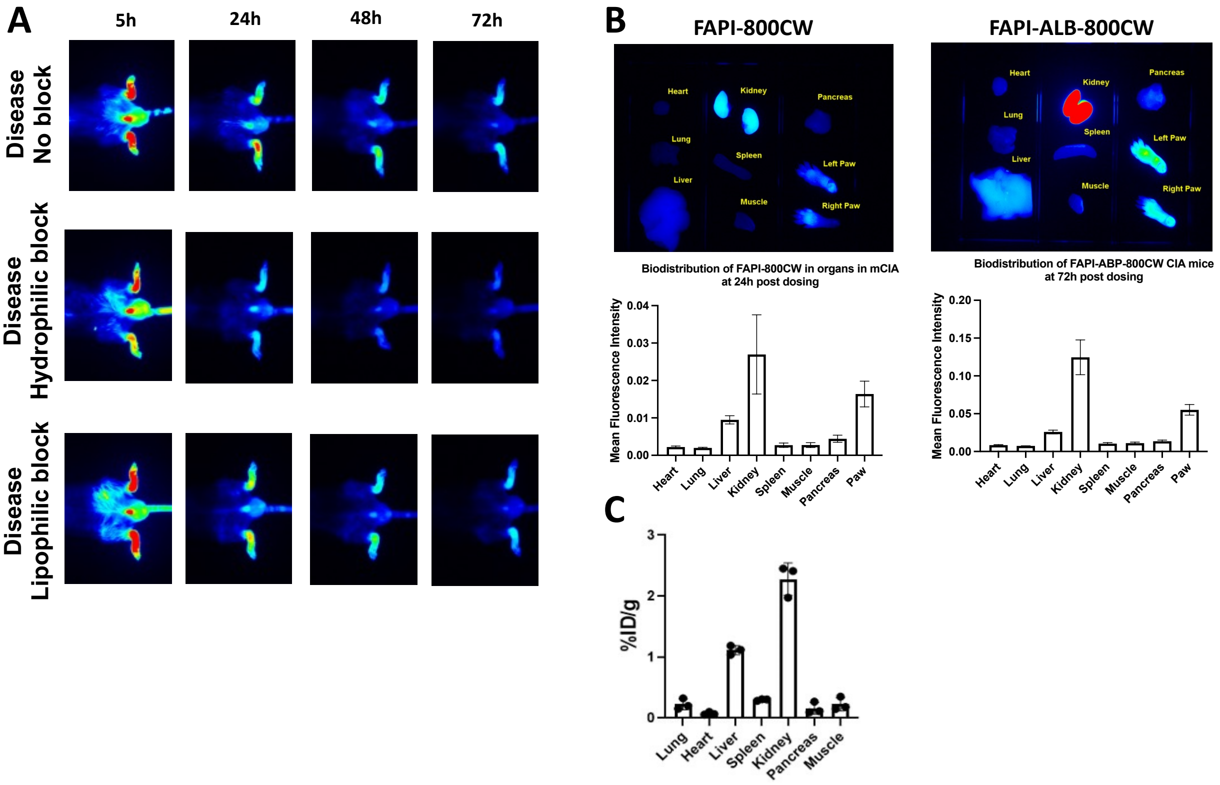

Figure S6. (A) Representative images illustrating longitudinal imaging of the slower clearing FAPI-ALB-800CW disposition in inflamed paws of the same CIA mouse up to 72 h post-dosing. The middle and bottom image panels demonstrate the temporal impact on the uptake with blocking with 50 nmol excess of FAPI-ALB-AF647 (hydrophilic) and FAPI-ABP (lipophilic), respectively. (B) Ex vivo biodistribution in mice dosed with FAPI-800CW (left) and FAPI-ALB-

800CW (right). The organ image panels, and the corresponding graphs below demonstrate limited uptake in all non-target organs, except kidney. The kidney uptake is related to the excretion of the labeled peptide. The uptake levels in the diseased paws are higher for both FAP reagents, consistent with images shown in (A) and in Figure 5. (C) Whole organ homogenate data are shown for FAPI-800CW at 24 h post-IV dose. The organs from this group were weighed, homogenized, and scanned with digest standards to quantify normalized uptake as described previously[4]. Aside from the high uptake in kidneys, consistent with the organ panel data in (B), the increased uptake observed in the liver may be due to a combination of increased negative charge density due to fluorophore conjugation and the residualizing nature of the fluorophore.

REFERENCES

(1) Lindner, T.; Altmann, A.; Krämer, S.; Kleist, C.; Loktev, A.; Kratochwil, C.; Giesel, F.; Mier, W.; Marme, F.; Debus, J.; Haberkorn, U. Design and Development of 99mTc-Labeled FAPI Tracers for SPECT Imaging and 188Re Therapy. *J. Nucl. Med. Off. Publ. Soc. Nucl. Med.* **2020**, *61* (10), 1507–1513. https://doi.org/10.2967/jnumed.119.239731.

(2) Bruncko, M.; Wang, L.; Sheppard, G. S.; Phillips, D. C.; Tahir, S. K.; Xue, J.; Erickson, S.; Fidanze, S.; Fry, E.; Hasvold, L.; Jenkins, G. J.; Jin, S.; Judge, R. A.; Kovar, P. J.; Madar, D.; Nimmer, P.; Park, C.; Petros, A. M.; Rosenberg, S. H.; Smith, M. L.; Song, X.; Sun, C.; Tao, Z.-F.; Wang, X.; Xiao, Y.; Zhang, H.; Tse, C.; Leverson, J. D.; Elmore, S. W.; Souers, A. J. Structure-Guided Design of a Series of MCL-1 Inhibitors with High Affinity and Selectivity. *J. Med. Chem.* **2015**, *58* (5), 2180–2194. https://doi.org/10.1021/jm501258m.

(3) Wai-Yee Leung, Shing-Ying Cheung, Stephen, Yue, PCT Int. Appl., **2002**, WO 2002026891 A1.

(4) Zhang, L.; Wang, Y.; Homan, K. T.; Gaudette, S. M.; McCluskey, A. J.; Chan, Y.; Murphy, J.; Abdalla, M.; Nelson, C. M.; Sun, V. Z.; Erickson, J. E.; Knight, H. L.; Clabbers, A.; Sterman, A. J. S.; Mitra, S. Imaging the Alternatively Spliced D Domain of Tenascin C in a Preclinical Model of Inflammatory Bowel Disease. *Mol Imaging Biol* **2022**. https://doi.org/10.1007/s11307-022-01758-6.
